# Screening for the prevention and early detection of cervical cancer: protocol for systematic reviews to inform Canadian recommendations

**DOI:** 10.1186/s13643-020-01538-9

**Published:** 2021-01-02

**Authors:** Allison Gates, Jennifer Pillay, Donna Reynolds, Rob Stirling, Gregory Traversy, Christina Korownyk, Ainsley Moore, Guylène Thériault, Brett D. Thombs, Julian Little, Catherine Popadiuk, Dirk van Niekerk, Diana Keto-Lambert, Ben Vandermeer, Lisa Hartling

**Affiliations:** 1 Alberta Research Centre for Health Evidence, University of Alberta, 11405 87 Avenue NW, Edmonton, Alberta T6G 1C9 Canada; 2 Department of Family and Community Medicine and Dalla Lana School of Public Health, University of Toronto, Toronto, Canada; 3 Centre for Chronic Disease Prevention and Health Equity, Public Health Agency of Canada, Ottawa, Canada; 4 Department of Family Medicine, University of Alberta, Edmonton, Canada; 5 Family Medicine, McMaster University, Hamilton, Canada; 6 Department of Family Medicine, McGill University, Montreal, Canada; 7 Faculty of Medicine, McGill University and Lady Davis Institute for Medical Research, Jewish General Hospital, Montreal, Canada; 8 School of Epidemiology and Public Health, University of Ottawa, Ottawa, Canada; 9 Faculty of Medicine, Memorial University, St. John’s, Canada; 10 Department of Pathology and Laboratory Medicine, The University of British Columbia, Vancouver, Canada

**Keywords:** Systematic review, Guideline, Uterine cervical neoplasms, Cervical intraepithelial neoplasia, Mass screening, Primary health care

## Abstract

**Purpose:**

To inform recommendations by the Canadian Task Force on Preventive Health Care on screening in primary care for the prevention and early detection of cervical cancer by systematically reviewing evidence of (a) effectiveness; (b) test accuracy; (c) individuals’ values and preferences; and (d) strategies aimed at improving screening rates.

**Methods:**

De novo reviews will be conducted to evaluate effectiveness and to assess values and preferences. For test accuracy and strategies to improve screening rates, we will integrate studies from existing systematic reviews with search updates to the present. Two Cochrane reviews will provide evidence of adverse pregnancy outcomes from the conservative management of cervical intraepithelial neoplasia. We will search Medline, Embase, and Cochrane Central (except for individuals’ values and preferences, where Medline, Scopus, and EconLit will be searched) via peer-reviewed search strategies and the reference lists of included studies and reviews. We will search ClinicalTrials.gov and the World Health Organization International Clinical Trials Registry Platform for ongoing trials. Two reviewers will screen potentially eligible studies and agree on those to include. Data will be extracted by one reviewer with verification by another. Two reviewers will independently assess risk of bias and reach consensus. Where possible and suitable, we will pool studies via meta-analysis. We will compare accuracy data per outcome and per comparison using the Rutter and Gatsonis hierarchical summary receiver operating characteristic model and report relative sensitivities and specificities. Findings on values and preferences will be synthesized using a narrative synthesis approach and thematic analysis, depending on study designs. Two reviewers will appraise the certainty of evidence for all outcomes using GRADE (Grading of Recommendations Assessment, Development and Evaluation) and come to consensus.

**Discussion:**

The publication of guidance on screening in primary care for the prevention and early detection of cervical cancer by the Task Force in 2013 focused on cytology. Since 2013, new studies using human papillomavirus tests for cervical screening have been published that will improve our understanding of screening in primary care settings. This review will inform updated recommendations based on currently available studies and address key evidence gaps noted in our previous review.

**Supplementary Information:**

The online version contains supplementary material available at 10.1186/s13643-020-01538-9.

## Background

### Description of condition and natural history of disease

Cervical cancer is a malignancy that affects the cells of the cervix, most commonly in the transformation zone where glandular cells of the endocervix transition to squamous cells of the exocervix [1, 2]. Persistent infection with human papillomavirus (HPV) is necessary, but not sufficient for the development of cervical cancer [3, 4]. Other factors that contribute to incidence or progression include immunosuppression, smoking, parity, and use of oral contraceptives [5]. Infection with high-risk HPV (hrHPV) genotypes is relatively common among sexually active individuals [6]. Among the routine screening population in Canada, pooled prevalence rates for the three most common HPV genotypes (16, 18, and 31) range from 3 to 47% [7]. Over their lifetime, most women (> 80%) and men (> 90%) will be infected with HPV, with the majority being infected before the age of 45 years [8]. Although about 90% of hrHPV infections resolve on their own within 2 years [9], others lead to slow and progressive changes to the cervix that can result in the development of cancer [6, 10, 11]. Among over 100 known HPV genotypes [9], 12 (genotypes 16, 18, 31, 33, 35, 39, 45, 51, 52, 56, 58, 59) have been designated as high-risk by the International Agency for Research on Cancer due to their strong oncogenic potential [12]. Among these, four are most commonly found within malignant cells of cervical cancer (genotypes 16, 18, 31, and 45). Genotypes 16 and 18 account for about 52% and 18% of cases of cervical cancer in Canada, with genotypes 31 and 45 accounting for 2% and 5% [13]. HPV genotype 16 has a high prevalence, high risk of progression, and low chance of clearance, making it overall the most high-risk genotype [14, 15].

**Fig. 1:**
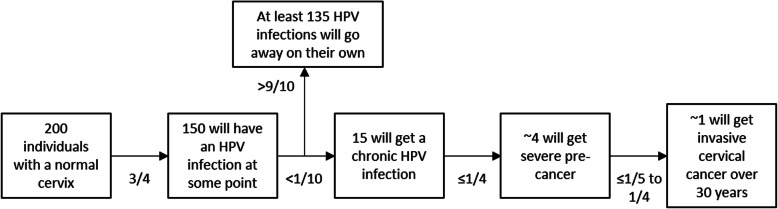
Progression from a human papillomavirus infection to invasive cervical cancer, assuming no treatment. Severe pre-cancer equates to CIN 3. Figures and proportions are approximates adapted from data reported in relevant literature [6, 8–11, 16–18]

The progression from HPV infection, to persistent infection, to pre-cancerous cervical changes, to invasive cervical cancer typically takes 10 to 15 years or more (Fig. 1) [9], but cases of more rapid progression less than 5 years have been reported [19]. Regression of pre-cancerous changes back to normal cervical cells is common especially among lower grade changes (i.e. low-grade squamous intraepithelial lesions (LSIL) or cervical intraepithelial neoplasia grade 1 (CIN 1)) and typically occurs within 1 to 2 years [20]. Although nearly all (~ 90%) newly acquired HPV infections will become undetectable within 1 to 2 years [20], the inability to detect the infection can represent either true viral clearance or immune control below detectable levels or viral latency [21–23]. A detectable immune response is generated approximately 60% of the time [24] (as evidenced by serum antibodies specific to the HPV genotype), resulting in questionable ability to provide immunity against re-infection [25]. At least in certain populations (e.g. immunosuppressed individuals), both reactivated and newly acquired infections can result in similar disease risk [25].

Different cytological reporting systems have been used to describe changes to cervical squamous cells identified during sampling (Table 1). The Bethesda system [30] refers to standardized cytology reporting on the adequacy of the sample and clinically relevant findings, specifically squamous intraepithelial lesions (SILs) graded as low or high grade. Changes to glandular cells are also described via the Bethesda system as atypical glandular cells (AGC) and adenocarcinoma in situ (AIS) [2, 30]. AGC are glandular cells with nuclear atypia that does not allow discernment between benign cells and in situ/invasive carcinoma. AIS is pre-invasive cervical adenocarcinoma that is confined to the epithelium and has not yet invaded through the basement membrane into deeper cervical tissue [2, 30]. In the UK, the British Society for Clinical Cytology (BSCC) ‘dyskaryosis’ terminology is used. In the BSCC system, cytological changes to glandular cells are graded based on the ratio of nuclear diameter to cytoplasmic diameter of dyskaryotic cells (i.e. those with an abnormal chromatin pattern) [26].

**Table 1:** Classification systems (adapted from the 2013 CTFPHC guideline review and other sources) [26–29]

Cytology			Histology	
**Dysplasia**	**Bethesda**	**BSCC**	**CIN**	**SIL**
Atypia	ASCUS	Borderline change in squamous cells	Atypia	
		Borderline change in endocervical cells		
HPV effect	LSIL	Low-grade dyskaryosis	HPV effect	LSIL
Mild dysplasia			CIN 1	
Moderate dysplasia	HSIL	High-grade dyskaryosis (moderate)	CIN 2	HSIL
Severe dysplasia		High-grade dyskaryosis (severe)	CIN 3	
Carcinoma in situ		High-grade dyskaryosis/?invasive squamous carcinoma		
Cancer	Cancer	?Glandular neoplasia of endocervical type	Cancer	AIS
		?Glandular neoplasia (non-cervical)		

*AIS* adenocarcinoma in situ, *ASCUS* atypical squamous cells of undetermined significance, *BSCC* British Society for Clinical Cytology, *CIN* cervical intraepithelial neoplasia, *HSIL* high-grade squamous intraepithelial lesion (also used for histological diagnoses CIN 2, CIN 3, and carcinoma in situ), *LSIL* low-grade squamous intraepithelial lesion, *SIL* squamous intraepithelial lesion

Pre-cancerous changes to the cervix may also be classified based on histology, rather than cytology. Specifically, in cervical intraepithelial neoplasia (CIN) terminology (Table 1), histological changes are graded as 1, 2, or 3 and/or cervical dysplasia (mild, moderate, severe, carcinoma in situ) [2, 31]. CIN may be suspected via cytological findings or colposcopic examination, but typically requires histopathological examination (via biopsy) for a definitive diagnosis [32]. SIL terminology may also be used to describe histological changes to the cervix (Table 1), whereby specimens that are positive for SILs are reported as either LSIL or high-grade SIL (HISL) [27]. SIL terminology may be further classified by the applicable CIN subcategorization, e.g. HSIL (CIN 2), based on clinical decision/management pathways [27]. SILs that cannot be graded due to limited sampling or other factors are reported as SILs, ungraded [27]. Specimens that are positive for endocervical glandular pre-invasive lesions are reported as AIS [27]. Specimens with some features of HSIL, AIS, or malignancy but for which definitive conclusions cannot be reached are reported as indeterminate for HSIL or AIS or malignancy [27].

If left untreated, many cervical lesions will regress or remain unchanged. In individuals with CIN 2 for example, within 2 years, about 50% (95% confidence interval (CI), 43 to 57%) will experience regression without treatment (either complete, i.e. normal histology or cytology results, or incomplete, i.e. CIN 1), 18% (95% CI, 12 to 39%) will experience progression to CIN 3 or worse (CIN 3+), and in 32% (95% CI, 23 to 42%), the lesion will persist (i.e. remain the same) [16]. Individuals with CIN 3 have a 25 to 30% risk of progression to invasive cervical cancer (ICC) over a 30-year period [17, 18].

### Burden of disease

As of 2020, cervical cancer is the 16th most commonly diagnosed cancer among Canadian adult females, with an age-standardized incidence rate of 4.1 per 100,000 population [33]. In 2020, it was estimated that 1350 Canadians would be diagnosed with ICC and that 410 would die from the disease [33]. The majority of cervical cancers are squamous cell carcinomas, i.e. cancers that begin in the squamous cells that cover the outer surface of the cervix (ectocervix) [2]. Squamous cell carcinoma most commonly develops in the transformation zone, where columnar cells are constantly being changed into squamous cells [2]. Most other cervical cancers are adenocarcinomas. Adenocarcinomas begin in the glandular cells that line the inside of the cervix (endocervix) [2]. Adenosquamous carcinomas, which affect both squamous and glandular cells, and other cervical tumours are less common [2]. Although earlier reviews found that cervical screening reduced the risk of cervical cancers generally, the risk reduction was greater for squamous cell carcinomas (risk ratio (RR) 0.46 (95% CI, 0.42 to 0.50) compared with adenosquamous carcinomas (RR 0.68 (95% CI, 0.56 to 0.82)) [34].

The incidence rate of cervical cancer varies by age. The median age of diagnosis of cervical cancer in Canada is 47 years, and just 28.7% of new cases are diagnosed in individuals under 40 [35]. Although the incidence among women in their twenties is relatively low (1.2/100,000 among those aged 20 to 24 years and 6.3/100,000 among those aged 25 to 29 years) [36], compared with other cancers of the reproductive system (i.e. uterine, ovarian), younger women are more likely to be diagnosed with cervical cancer [35]. At the highest risk are those in their early forties, with an incidence rate of 16.6/100,000 among those aged 40 to 44 years [35]. Between 2011 and 2015, the majority of new cervical cancers in Canada were diagnosed at an early stage of disease (stage I, 54.4%), while 11.8% were diagnosed with advanced (stage IV) disease [37]. The percentage of cases diagnosed at stage I is smaller with increasing age (18–24 years, 78.6%; 25–39 years, 71.5%; 40–54 years, 53.8%; 55–69 years, 40.5%; 70+ years, 26.6%), whereas the percentage of cases diagnosed at stage IV is higher in older individuals (18–24 years, 0.0%; 25–39 years, 4.6%; 40–54 years, 11.5%; 55–69 years, 17.7%; 70+ years, 22.6%) [37]. Five-year net survival from cervical cancer in Canada is about 73% [37]. Survival is much higher for localized cancers (stage I, 86%) compared with cancers diagnosed with regional involvement (stage II or III, 56%) or with distant metastases (stage IV, 17%) (US data, where five-year net survival is about 63%) [38], indicating lower mortality with less advanced cancer.

### Prophylactic HPV vaccination

The first HPV vaccine in Canada was approved in 2006 [39]. Currently, two HPV vaccines are available: bivalent (Cervarix™ or HPV2) and nine-valent (Gardasil 9® or HPV9) [40]. Both vaccines protect against HPV genotypes 16 and 18 [40]. The HPV9 vaccine protects against five additional hrHPV genotypes (31, 33, 45, 52, and 58) [40]. HPV vaccination is offered on a 2- or 3-dose schedule depending on age [40]. Research investigating the efficacy of a 1-dose schedule is underway [41].

In a systematic review of 26 trials (73,428 participants) with 3.5 to 8 years of follow-up, Arbyn et al. [42] reported high-certainty evidence that HPV vaccines given to adolescent girls and women aged 15 to 26 years reduce the risk of pre-cancerous cervical lesions (from 164 to 2 (95% CI, 0 to 8) cases per 10,000 for CIN 2 (3 trials, 23,676 participants) and from 70 to 0 (95% CI, 0 to 7) cases per 10,000 for CIN 3+ (2 trials, 20,214 participants) associated with HPV 16 or 18). The prophylactic effect was greatest for HPV genotypes 16 and 18, and among those negative for hrHPV or HPV genotypes 16 and 18 at baseline. For adolescent girls and women aged 15 to 45 years who were negative for HPV genotypes 16 and 18, there was moderate-certainty evidence that HPV vaccines reduce the risk of CIN 2+ from 45 to 14 (95% CI, 5 to 37) cases per 10,000 (2 trials, 7552 participants) [42]. In a separate systematic review (20 trials; 31,940 participants), Bergman et al. [43] found moderate- to high-certainty evidence that 2- and 3-dose schedules of HPV vaccines in young females induce comparable immunogenicity, based on HPV antibody response. There was high-certainty evidence that quadrivalent and nine-valent vaccines result in similar protection against cervical, vaginal, and vulval pre-cancers and cancers [43].

All provinces and territories in Canada have publicly funded, gender-neutral, school-based HPV vaccination programmes [44]. Publicly funded administration of HPV vaccine was implemented in school-aged girls between 2007 and 2009 (except Nunavut, where it was implemented in 2013) and in school-aged boys between 2013 and 2017 [44]. The most recent coverage data show that among girls, the immunization uptake for the final dose ranges from 57% (Northwest Territories) to 92% (Newfoundland and Labrador). Among boys, the immunization uptake for the final dose ranges from 53% (Ontario) to 90% (Prince Edward Island) [44].

The changing epidemiology of HPV infection following the introduction of prophylactic vaccines [45, 46] has implications for the relative benefits and harms of screening, test accuracy, and patients’ values and preferences [47]. At the current rate of coverage among boys and girls in 2018, it was projected that 3395 cases of cervical cancer could be prevented by 2032 [48]. In the context of high vaccine coverage over a sufficient period of time, the pre-cancerous lesions targeted by cervical screening tests may become so rare that the harms from screening may outweigh its benefits [49]. As the prevalence of cervical lesions diminishes, population-based screening using cytology may become very inefficient [47, 49] and the predictive value of cytological screening tests will be reduced [50]. Future screening guidelines will need to consider vaccine uptake and the prevalence of HPV infection. Dependent on the local context, there may be interest in personalizing screening based on vaccination status [51].

### Screening for cervical cancer

The initial test developed for use in cervical screening was the Papanicolaou (Pap) test. The test, which can detect pre-cancerous abnormalities in cells collected from the cervix, was first introduced in Canadian centres as local trials in the late 1940s and 1950s [52]. More recently in the 1990s, the strong causal association between persistent infection with hrHPV genotypes and cervical cancer led to the development of cervical screening tests that detect HPV DNA and RNA [53]. Randomized controlled trials (RCTs) have investigated the use of hrHPV tests, alone as the primary screening tool for cervical cancer [54–57], for co-testing with cytology [58–61], and followed by various forms of triage [62]. Long-term follow-up of women enrolled in these trials is ongoing; thus, evidence of the benefits and harms of hrHPV testing requires continuing review.

Unlike the Pap test, cervical samples for the hrHPV test can be self-collected (either at home or at a primary care centre), which has the potential to reduce barriers to screening; however, in previous reviews, authors have suggested that further evidence on the agreement of findings between self- and clinician-sampled tests is required before recommendations on the use of self-collected samples are made [63]. Urine-based sampling for hrHPV is less invasive and potentially more acceptable to patients, but the reported accuracy of the approach varies substantially across studies [64]. Urine-based testing is not approved for cervical screening in Canada. Previous review authors have suggested that to adopt the test into practice, testing methods must be more consistent and reproducible [64].

High-risk HPV tests may also have a lower specificity than cytology resulting in higher rates of false positives and potentially unnecessary colposcopies (those that test for DNA more so than those that test for RNA) [65–67]. Because hrHPV testing is not yet offered in many Canadian jurisdictions [44], its adoption within organized cervical screening programmes would require changes to laboratory configuration, workflow, and human resources.

In Canada, all provinces except Quebec have organized cervical screening programmes. As defined by an expert group of the International Union Against Cancer, organized screening programmes have the following: (1) a defined and identifiable target population; (2) strategies to ensure high coverage (e.g. personal invitations with times and places for screening); (3) adequate facilities for taking screening tests and laboratory services to examine them; (4) quality control programmes for taking and interpreting screening tests; (5) adequate facilities for diagnosis, treatment, and follow-up of abnormal tests; (6) an established referral system to help facilitate individuals through the screening process; and (7) organized evaluation and monitoring of the impact of the programme with established data quality control programmes [68, 69]. In Canada, these programmes are typically organized at the provincial level and are generally focused on individuals who do not have signs or symptoms of cervical cancer. In Quebec as well as Nunavut, Yukon, and the Northwest Territories, opportunistic screening is offered by primary care providers (plans are underway to implement an organized screening programme in the Yukon Territory) [44]. Five jurisdictions (Alberta, Saskatchewan, Manitoba, Ontario, and New Brunswick) use initial letters of invitation as a recruitment method for never-screened women [70]. The letters provide information on screening and eligibility and invite women to participate in screening [70]. In Newfoundland and Labrador, a letter of invitation is pending implementation, and other recruitment methods include generating a routine recall list for primary care providers [70]. In Nunavut, phone calls are used for recruitment. Other jurisdictions do not use standardized recruitment methods [70].

In eight jurisdictions (Nunavut, British Columbia, Alberta, Saskatchewan, Manitoba, Ontario, New Brunswick, and Newfoundland and Labrador), if the screening result is normal, participants and/or their primary care providers receive a recall telephone call and/or letter at a pre-defined interval [70]. In the case that a screening result is abnormal, the participant and/or their primary care provider is sent a letter of notification [70]. Individuals with abnormal results may have repeat cytology testing or HPV triage, or be referred directly for colposcopy, for evaluation and biopsy, the exact pathway and algorithm varying by jurisdiction [70]. Those identified via follow-up testing as having pre-cancerous lesions or ICC are referred for appropriate management.

Preferences for or against a screening strategy can be a consequence of the relative importance people place on the expected or experienced outcomes [71]. Despite the anticipated benefits from cervical cancer screening, including the early detection and treatment of pre-cancerous lesions, potential harms exist, including frequent follow-up testing and invasive diagnostic procedures (e.g. biopsy, colposcopy), unnecessary treatment of false-positive results, and psychological harms associated with positive tests [72]. As many pre-cancerous lesions will never become clinically important over an individual’s lifetime, overdiagnosis of such lesions is of concern for patients and providers as it can lead to unnecessary testing and treatment and the harms associated with these procedures.

In Canada, 74% of women aged 25 to 69 years receive a Pap test every 3 years [73]; however, some population subgroups, including Indigenous populations [74], individuals with very low socioeconomic status [75], individuals living in rural or remote communities, new immigrants, people with a history of trauma or abuse [76], imprisoned individuals [77], and other underserved groups [78] are more likely to be inadequately screened. Transgender individuals (e.g. female-to-male transgender men) have also been identified as a group at risk for inadequate cervical cancer screening [79]. There is a need to evaluate the effectiveness of interventions aimed at improving screening rates, especially among under- and never-screened populations.

### Rationale and scope of systematic review

At present, screening programmes for the prevention and early detection of cervical cancer in provinces and territories use cytology-based screening methods using the Pap test. Planning for primary hrHPV testing is underway in Ontario and its use is under consideration in British Columbia and Quebec. Several provinces and territories have also started to implement or pilot test hrHPV testing for triage [70]. In 2013, the Canadian Task Force on Preventive Health Care published a guideline on screening for the prevention and early detection of cervical cancer which recommended women aged 25 to 69 years be screened every 3 years with Pap testing; women aged 24 years and younger not be routinely screened; and women aged 70 years or older, who have undergone adequate screening, not be screened [80]. Uptake of these recommendations across the country has been mixed, with most provinces and territories initiating screening at age 21 years, with the exception of British Columbia, Alberta, and Prince Edward Island where screening has recently been revised to follow the Task Force recommendation of starting at age 25 years [44, 81].

The 2013 Task Force screening guidelines were limited to cytological screening for the prevention and early detection of cervical cancer. At the time, the Task Force felt it was premature to make recommendations on the use of hrHPV testing due to the limited evidence identified; this was identified as a gap that should be addressed as more evidence became available. Since the release of the 2013 guideline, more recent international guidelines (including Australia, the UK, the Netherlands, and the USA) have provided recommendations on the use of hrHPV testing in cervical cancer screening [72, 82–84]. New studies have also been published that are likely to improve our understanding of screening in primary care settings for the prevention and early detection of cervical cancer. Thus, we will undertake several systematic reviews to inform an update of the 2013 Task Force guideline. Specifically, we aim to identify and synthesize evidence on the following:

- the effectiveness (benefits and harms) and comparative effectiveness of various cervical screening strategies;
- the comparative accuracy of various screening tests and strategies;
- values and preferences for outcomes from cervical screening; and
- the effectiveness of interventions aimed at improving screening rates in under-screened and never-screened individuals.

## Methods

### Systematic review conduct

The Evidence Review and Synthesis Centre (ERSC) at the University of Alberta (AG, JP, DK-L, BV, LH) will conduct the systematic reviews on behalf of the Task Force following the research methods outlined in the Task Force methods manual [85]. We will follow a pre-defined protocol, reported in accordance with current standards (Supplementary File 1) [86], as documented herein. During protocol development, a working group was formed consisting of Task Force members (DR, CK, AM, GTh, BDT), with input from clinical experts (JL, CP, DvN), and scientific support from the Global Health and Guidelines Division at the Public Health Agency of Canada (RS, GTr). The working group contributed to the development of the key questions (KQs) and PICOTS (population, intervention(s) or exposure(s), comparator(s), outcomes, timing, setting, and study design) elements.

Task Force members made the final decisions with regard to the KQs and PICOTS. Task Force members and clinical experts rated the proposed outcomes based on their importance for clinical decision-making, according to methods of Grading of Recommendations Assessment, Development and Evaluation (GRADE) [87]. Ratings by the clinical experts were solicited to ensure acceptable alignment with the views of Task Force working members (clinical decision-makers), but Task Force members determined the final ratings. Final critical outcomes (rated at 7- or above on 9-point scale) pertaining to the effectiveness and comparative effectiveness of screening included the following: the rate of ICC, cervical cancer mortality, all-cause mortality, the rate of CIN 2 and CIN 3, and overdiagnosis of CIN 2, CIN 3, and ICC. Final important outcomes (rated 4–6) for inclusion were as follows: the number and rate of colposcopy and/or biopsy (or referral rate), adverse pregnancy-related outcomes from conservative management of CIN, and the false-positive rates for detecting CIN 2, CIN 3, and ICC. These outcomes are defined in Supplementary File 2. Other outcomes relevant to comparative accuracy, values, preferences, and the effectiveness of interventions to improve screening rates were selected by the Task Force working members in collaboration with the ERSC. The classification of benefit or harm for all outcomes will be based on the effects observed for different comparisons.

This version of the protocol was reviewed by the entire Task Force. Stakeholders (*n* = 17) reviewed a draft version of this protocol, and all comments were considered. Throughout the conduct of the systematic reviews, we will document any changes to the protocol (including timing), with justification. We will report on these within the final report. We will report our findings in accordance with available standards at the time of writing (i.e. v. 2009 [88] or updated version of the PRISMA (Preferred Reporting Items for Systematic Reviews and Meta-analyses) Statement, should it become available prior to submission of the final report).

### Key questions and analytical framework

The Task Force has delineated five KQs to inform their recommendations, as follows:

- KQ 1: What are the effectiveness (benefits and harms) and comparative effectiveness of different screening strategies for the prevention and early detection of cervical cancer?
- KQ 1a: Do the effectiveness and comparative effectiveness of different screening strategies for the prevention and early detection of cervical cancer differ by age or by other population subgroups?
- KQ 2: What is the comparative accuracy of screening tests for the prevention and early detection of cervical cancer?
- KQ 2a: Does the comparative accuracy of screening tests differ by age or by HPV vaccination status?
- KQ 3: What are the adverse pregnancy outcomes associated with conservative management of CIN? (NB. will not require a new or updated systematic review)
- KQ 4: What is the relative importance individuals place on the potential outcomes from screening for the prevention and early detection of cervical cancer?
- KQ 5: What is the effectiveness of primary care-based interventions to increase rates of screening for the prevention and early detection of cervical cancer for under- and never screened individuals?

**Fig. 2:**
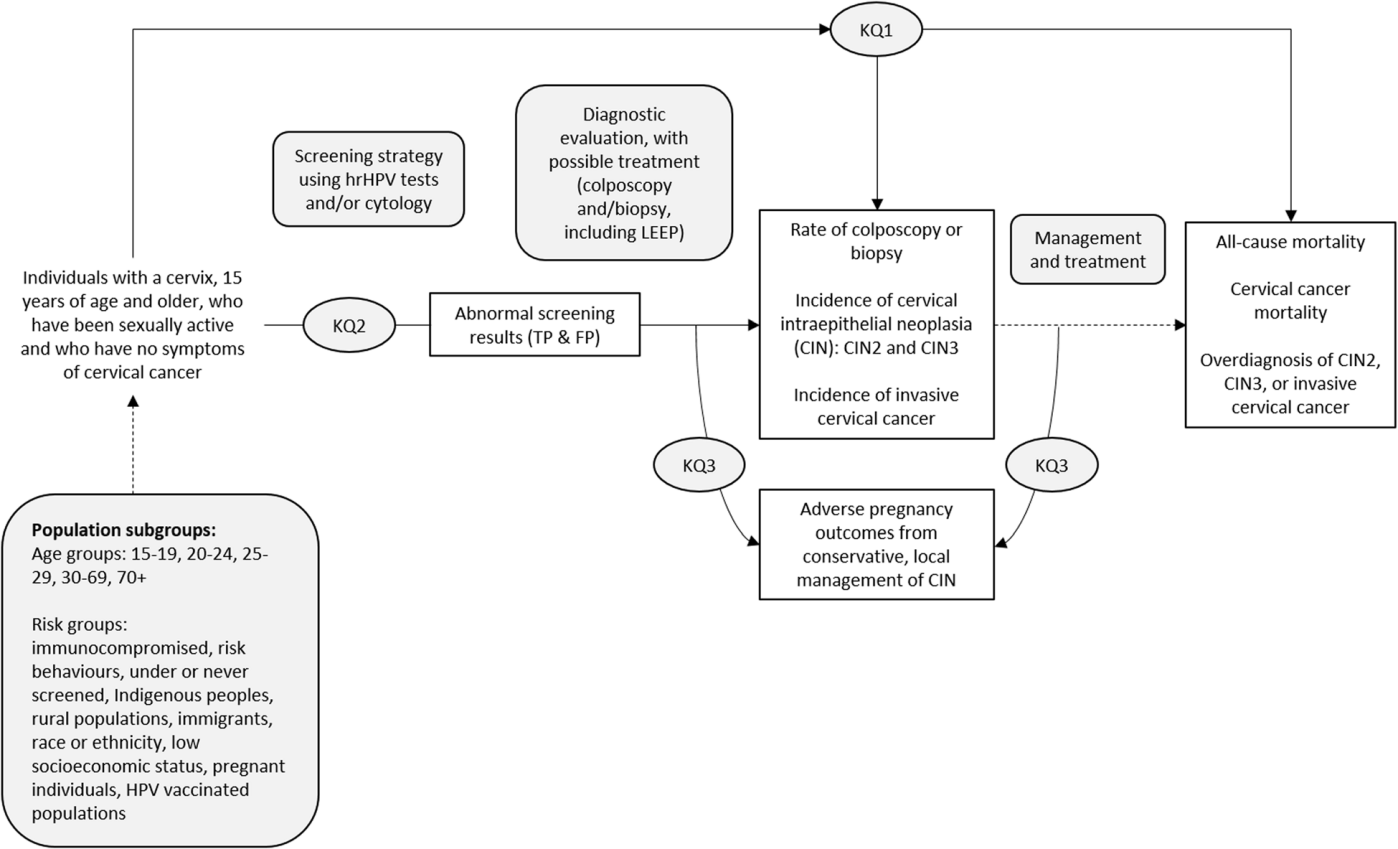
Analytical framework

For the purpose of these reviews, we will consider effectiveness to include both benefits and harms. The analytical framework in Fig. 2 shows the population (and population subgroups), KQs, and outcomes in the context of the screening, diagnosis, management, and treatment modalities under consideration.

The systematic reviews for KQs 1 and 2 focus on the effectiveness and comparative effectiveness (KQ 1) and the comparative accuracy (KQ 2) of various screening strategies. The intent for KQ 2 is to fill gaps for the outcomes from KQ 1. The main goal is to compare detection rates and harms (i.e. false positives, false negatives) between different screening strategies, and to provide indirect evidence for KQ 1 with respect to false-positive rates, as we expect evidence for this outcome to be of low or very low certainty from studies contributing to KQ 1. It may also provide information about the comparative accuracy of screening tests not studied in KQ 1 to help determine if these may be appropriate to use in practice in the absence of KQ 1 evidence.

KQ 3 focuses on the adverse pregnancy outcomes (the only direct treatment or management harm rated as important by the working group) associated with conservative management of CIN 2 and CIN 3. The intent of this KQ is to fill gaps for adverse pregnancy outcomes identified in the studies for KQ 1. The rationale for a separate KQ is that adverse pregnancy outcomes are unlikely to be reported in studies focusing primarily on screening effectiveness. In the United States Preventive Services Task Force (USPSTF) 2018 review of screening for cervical cancer with hrHPV testing [67], none of the included screening trials (*n* = 8) [54–61, 89–99] reported on adverse pregnancy outcomes.

The ERSC will not undertake de novo searches or syntheses for KQ 3. During protocol development, a research librarian undertook a comprehensive search of existing systematic reviews published between 2014 and March 2019. These systematic reviews were scrutinized for suitability, with careful consideration for the comprehensiveness of their searches, scope (i.e. ability to capture the studies of interest), and reporting quality. We identified two Cochrane systematic reviews, published in 2015 [100] and 2017 [101], that answer our KQ 3. The Cochrane Review Group has confirmed that both reviews are presently being updated to incorporate the latest evidence, and these reviews will be used by the Task Force.

Of the two Cochrane reviews, the review by Kyrgiou et al. published in 2015 [100] synthesized evidence on fertility and early pregnancy outcomes (i.e. pregnancy rates, miscarriage rates, ectopic pregnancies) following conservative excisional or ablative management of CIN. Fifteen observational studies (> 2 million participants) were included. The review by Kyrgiou et al. published in 2017 synthesized evidence on the obstetric outcomes (i.e. preterm birth, low birth weight, cervical cerclage) following conservative excisional or ablative management of CIN. Sixty-nine observational studies (> 6 million participants) were included. Due to the observational study design of the available evidence, both reviews reported very low- to low-certainty evidence for the effects of the interventions for our outcomes of interest. Given that it would be unethical to conduct RCTs to address this question (i.e. where women with CIN would be randomized to a non-treatment control group), the probability of identifying a newly published trial that will improve the certainty of evidence for the outcomes of interest is assessed as virtually zero. Additional observational evidence is also unlikely to improve the certainty of evidence, but could impact the pooled effect estimates. As the two systematic reviews are presently undergoing updates, to avoid duplication of research effort, the Task Force will rely on these two reviews to inform KQ 3. The ERSC will review, contextualize, and summarize the available evidence (i.e. in text, tables, and figures) from the two Cochrane systematic reviews to facilitate interpretation by the Task Force during guideline development.

The review for KQ 4 will synthesize evidence of the relative importance individuals place on the outcomes from cervical screening (independent of the screening strategy) [102, 103], including all critical and important outcomes as defined for KQ 1 (Table 2). It will also provide information to the Task Force on whether there is important uncertainty about or variability in how much people value the main outcomes [102].

**Table 2:** Eligibility criteria for key question 1 (effectiveness and comparative effectiveness)

Criterion	Inclusion	Exclusion
Population	Individuals with a cervix, 15 years of age and older, who have been sexually active, and who have no symptoms of cervical cancer^a^ Population subgroups: – By age group (15–19, 20–24, 25–29, 30–69, 70+) – Risk groups: immunocompromised (e.g. HIV, organ transplantation, chemotherapy or chronic use of corticosteroids, use of disease-modifying anti-rheumatic drugs or biologics); risk behaviours (e.g. early sexual debut, women who have sex with women, individuals who have multiple sexual partners, smoking); under or never screened (e.g. transgender individuals, individuals with a history of trauma or abuse); Indigenous peoples; rural populations; immigrants; race or ethnicity; low socioeconomic status; pregnant individuals; HPV-vaccinated populations	Study population includes > 25% individuals with recent abnormal screening result
Intervention	Any screening strategy using hrHPV tests and/or cytology with subsequent follow-up of abnormal tests: – Primary screening with cytology (conventional or liquid-based) – Primary screening with hrHPV testing – Cytology screening, which if abnormal may be followed by triage with an hrHPV test – hrHPV screening, which if positive may be followed by triage with cytology or other hrHPV test (e.g. full genotyping) – Other combinations will be considered	HPV test using in situ hybridization, p16 immunostaining, or HPV viral load Urine for sample collection Point-of-care tests Co-testing as a strategy (although we will include relevant data for the individual strategies where suitable)
Comparator	Effectiveness: No routine screening Comparative effectiveness: Any screening strategy differing by one or more of the following factors: – Screening test strategy – Screening interval – Universal vs. selective/targeted (e.g. starting age) – Method of sample collection (e.g. self-collection^b^ (self-collection at home vs. self-collection in clinic) vs. health provider collection) – Protocol for evaluation of abnormal screening results (e.g. criteria for immediate colposcopy)	
Outcomes	Critical outcomes: – Incidence of invasive cervical cancer (squamous and adenocarcinoma) – Incidence of cervical intraepithelial neoplasia (CIN) 2 and CIN 3^c^ – Cervical cancer mortality – All-cause mortality – Overdiagnosis of CIN 2 and CIN 3 and invasive cervical cancer^c^ Important outcomes: – Number and rates of colposcopy and/or biopsy, including LEEP and other treatments provided during colposcopy (or referral rate) (for comparative effectiveness) – Adverse pregnancy outcomes from conservative, local management of CIN – False-positive rate for detecting CIN 2 and CIN 3 and invasive cancer^c^	
Timing	No limitation on the duration of follow-up; results will be reported by screening round and longest follow-up	
Setting	Studies from Very High Human Development Index countries	
Study design	– Randomized controlled trials – If insufficient data from randomized controlled trials (by comparison and outcome): non-randomized studies (controlled trials, before-after studies, interrupted time series, individual patient data meta-analysis, cohort studies, case-control studies)	Conference proceedings; government reports; systematic reviews; case reports; editorials
Language	English or French	
Publication date	1995–present	

*CIN* cervical intraepithelial neoplasia, *HIV* human papillomavirus, *LEEP* loop electrosurgical excisional procedure

^a^We will include studies where up to 25% of the participants had a recent abnormal screening result

^b^Different samples or methods of sample collection

^c^The ability to report and analyze findings by CIN 2, CIN 3, and invasive cervical cancer will be determined after reviewing the outcomes used in the identified studies (e.g. CIN 2+ and CIN 3+ will be considered if necessary and may be considered indirect)

Given that certain Canadian subpopulations remain under-screened or never-screened despite recommendations for cervical screening, the review for KQ 5 will inform primary care interventions that may improve screening rates.

### Eligibility criteria

Tables 2, 3, 4, and 5 show the PICOTS elements for KQs 1, 2, 4, and 5. These are described in detail in Supplementary File 3. Given that we will not undertake de novo synthesis for KQ 3, we have not included PICOTS elements for this KQ.

**Table 3:** Eligibility criteria for key question 2 (comparative diagnostic accuracy)

Criterion	Inclusion	Exclusion
Population	Individuals with a cervix, 15 years of age and older, who have been sexually active, and who have no symptoms of cervical cancer^a^ Population subgroups: – By age group (15–19, 20–24, 25–29, 30–69, 70+) – HPV-vaccinated populations	Study population includes > 25% individuals with recent abnormal screening results
Index screening test	– Primary high-risk HPV testing with HPV nucleic acid tests^b^ alone – High-risk HPV testing with HPV nucleic acid tests, followed by some form of triage (e.g. cytology or HPV testing with partial genotyping for HPV 16 or 18, sequential partial genotyping for HPV 16 or 18 followed by cytology to further triage those positive for HPV 16 or 18). Subgroups: – Method of sample collection for high-risk HPV testing (i.e. self-collected (home vs. in clinic) vs. clinician-collected) – Type of assay (i.e. generic, partial genotyping, full genotyping) – HPV test threshold for a positive result (i.e. 1 pg/mL, 2 pg/mL)	HPV test using in situ hybridization, p16 immunostaining, or HPV viral load Earlier versions of commercial tests that have been replaced (e.g. Hybrid Capture 1) Urine for sample collection Point-of-care tests
Comparator screening test	– Conventional or liquid-based cytology, with or without follow-up by high-risk HPV testing – High-risk HPV testing with HPV nucleic acid tests, followed by different forms of triage than in the index test – hrHPV testing with HPV nucleic acid tests, using a different method of sample collection (i.e. self-sampled (home vs. clinic) vs. clinician-sampled)	Visual inspection with acetic acid or visual inspection with Lugol’s iodine
Reference standards	• Colposcopy with histologic examination of tissue specimens, when indicated. • Study protocol stipulates that reference standard is applied to: – All patients, or – All screening test-positive patients and a subset (e.g. random 10%) of screening test-negative patients	Reference standard only applied to screen-positive patients
Outcomes and target conditions	Diagnostic test accuracy: Number and proportion of people positive and negative on each test (TP, FP, TN, FN), sensitivity and specificity to screen for high-grade cervical lesions (CIN 2, CIN 3, HSIL), and/or invasive cervical cancer (squamous cell carcinoma or adenocarcinoma)	
Timing of reference standard	Reference standard test performed before any management based on the index test result	
Setting	Studies from Very High Human Development Index countries	
Study design	– Observational studies (e.g. prospective or retrospective cohorts, or cross-sectional studies) in which all participants receive both the index and comparator screening test, followed by verification of disease status using the reference standard in all patients or in all screening test-positive patients and a subset (e.g. random 10%) of screening test-negative patients – Randomized controlled trials where participants are randomized to different screening tests but all receive the same reference standard	Conference proceedings; government reports; systematic reviews; case reports; case-control studies; editorials
Language	English or French	
Publication date	1995–present	

*AGC* atypical glandular cells, *AIS* adenocarcinoma in situ, *CIN* cervical intraepithelial neoplasia, *FN* false negative, *FP* false positive, *HPV* human papillomavirus, *HSIL* high-grade squamous intraepithelial lesions, *TN* true negative, *TP* true positive

^a^We will include studies where up to 25% of the participants had a recent abnormal screening result

^b^Eligible HPV tests include generic assays, as well as partial and full genotyping assays able to detect at least some high-risk HPV genotypes (e.g. HPV 16, 18, 31, 33, 35, 39, 45, 51, 52, 56, 58, 59, 68) and available commercially in Canada or reasonably perceived to potentially be available in Canada. Examples of eligible high-risk HPV tests include the Cobas 4800 HPV Amplification/Detection Kit (Roche Molecular Systems, Inc.), Linear Array HPV Genotyping Test (Roche), Aptima HPV assay (Hologic, Inc.), Aptima HPV 16 18/45 Genotype Assay (Hologic), Cervista HPV HR assay (Hologic), Abbott RealTime High-Risk HPV (Abbott Molecular), Digene DML-2000 HPV Test Hybrid Capture 2 (Qiagen Sciences LLC), and Xpert HPV test (Cepheid)

**Table 4:** Eligibility criteria for key question 4 (patient values and preferences)

Criteria	Inclusion	Exclusion
Population	Individuals with a cervix, or who have had their cervix removed as part of treatment for cervical cancer, 15 years of age and older (patients and the general public) Population subgroups: – Age (15–19, 20–24, 25–29, 30–69, 70+) – Risk groups: immunocompromised (e.g. HIV, organ transplantation, chemotherapy or chronic use of corticosteroids, use of disease-modifying anti-rheumatic drugs or biologics), risk behaviours (e.g. early sexual debut, women who have sex with women, individuals who have multiple sexual partners, smoking), Indigenous peoples, rural populations, immigrants, lower SES, pregnant individuals, HPV-vaccinated populations – Previous screening history (regular as per guidance vs. not regular (under) vs. never-screened)	
Exposures	– Experience with critical outcome(s) related to screening or, – Exposure to clinical scenarios or information about potential critical outcomes and/or estimates of effect on outcome risks from screening, or – No experience or exposure to information about outcomes, but authors are soliciting probability trade-offs or ratings of different potential critical outcomes (e.g. number of biopsies acceptable to prevent one early diagnosis of invasive cervical cancer) – Focus of study is on consideration of possible, or assessment of experienced, outcomes from screening. Exposure moderators: differing descriptions or experience of outcomes in terms of stage, treatments received, severity, time since diagnosis (immediate vs. first year vs. later years); number of outcomes considered; differing estimates of magnitudes of effect from screening (if applicable)	Apart from studies with direct (e.g. time-trade off) or indirect (e.g. based on EQ-5D) measurement of heath state utilities, participants need to consider at least one outcome that may be a harm from screening (e.g. false positives, overdiagnosis [e.g. hrHPV+ but never will get cancer], increased CIN 2+ detection). Focus on the harms from management of lesions or cancer.
Comparisons	– Different critical outcome or groups of outcomes (e.g. critical benefits vs. harms) – Healthy state without outcome (for utility studies) – No comparison (for utility studies) – No or another intervention, if applicable for interpreting outcome importance, i.e. no screening, another screening strategy (e.g. having different magnitude of effects), no information (e.g. in studies using decision aids). When only one arm (e.g. receiving decision aid) of a comparative study is used for interpreting data on patient preferences, the study will be classified as a non-comparative study.	
Outcomes	a) Utility values/weights for the potential outcomes from screening b) Non-utility, quantitative information about relative importance of different outcomes (e.g. rating scales using ordinal or interval variables, ranking; preference for or against screening [screening attendance, intentions, or acceptance] or preferred screening strategy based on different outcome risk descriptions, strength of associations about outcome ratings with screening behaviours or intentions) c) Qualitative information indicating relative importance between outcomes d) Rank-order of importance of outcomes, based on data from a) to c) above, as applicable. Data must relate to the outcomes considered critical to the Task Force. Outcome groupings a) to c) above will be included in a hierarchical manner for each critical screening outcome.	
Timing	Follow-up duration: any or none	
Setting	Any setting in Very High Human Development Index countries	
Study design and publication status	Any quantitative or qualitative study design using the methods described below: – Utility values/weights measured directly using time trade-off^a^, standard gamble^b^, visual analogue scales, conjoint analysis with choice experiments or probability trade-offs – Utility values/weights measured or estimated indirectly, e.g. from transforming several health state domains from multi-attribute utility indexes such as EQ-5D to utilities using general population preferences, including mapping from generic or disease-specific health-related quality of life instruments – Non-utility, quantitative information about relative importance of different outcomes, e.g. rating scales using ordinal or interval variables, ranking; preference for or against screening (screening attendance, intentions, or acceptance) or preferred screening strategy based on different outcome risk descriptions, strength of associations about outcome ratings with screening behaviours or intentions) – Qualitative information indicating relative importance between benefits and harms – Rank-order of importance of outcomes	Conference proceedings; government reports; editorials
Language	English or French	
Publication date	2000–present	

*HPV* human papillomavirus

^a^Time trade-off measures the value placed on attributes of a commodity by requiring individuals to choose between different scenarios, where in each scenario, the commodity in question has varying levels of different attributes

^b^Standard gamble approaches require that respondents choose between a lifetime in a certain health state or a gamble between different health states, whereas time trade-off requires respondents to choose between living for a period in less than perfect health, as opposed to a shorter period in perfect health

**Table 5:** Eligibility criteria for key question 5 (interventions to increase screening rates)

Criterion	Inclusion	Exclusion
Population	Individuals with a cervix who would meet the criteria for cervical cancer screening, but who have never been screened or who have been under-screened, as defined by the study authors, when assessed against current screening recommendations (e.g. for screening interval). Population subgroups: – Indigenous peoples – Immigrant groups – Rural populations – Low socioeconomic status populations	Individuals with symptoms of cervical cancer or previous abnormal test results on cervical screening (unless cleared to return to normal screening) Individuals who have had complete surgical removal of the cervix
Intervention	– Mail-out or opt-in (invitation to request) self-sampling for hrHPV screening – Other interventions aimed at individuals or primary care providers with the intent to increase acceptability of screening (e.g. screening reminders, education, counselling, provider recommendation, addressing cultural practices and beliefs, patient-provider communication)	Interventions not targeted to primary care providers or feasible for primary care to deliver to their patients (e.g. community or lay health workers, community distribution of HPV self-sampling kits)
Comparator	– No intervention – Routine care (could include reminders or invitations to screen, or other forms of minimal intervention like pamphlets, posters)	
Outcomes	Screening rate	
Timing	No limitation on the duration of follow-up	
Setting	– Primary care settings or settings available through primary care referral (note we will not exclude primary care interventions that are implemented alongside or in support of broader public health initiatives (e.g. reminders)) – Studies involving populations from Very High Development Index countries	
Study design	– Randomized controlled trials – Non-randomized trials and cohort studies (will only be considered if there are no data available from randomized controlled trials)	Conference proceedings; government reports; case series; case reports; case-control studies; editorials
Language	English or French	
Publication date	2000–present	

*HPV* human papillomavirus

### Efficiencies by integrating or using existing systematic reviews

Where possible, we will either update one or more existing systematic reviews or, if we are not aware of systematic reviews that are good candidates for an update, integrate studies from existing systematic reviews [104]. When available, we may use existing high-quality, up-to-date systematic reviews as is without de novo searches or syntheses if they align well with the scope of our KQs and PICOTS elements (fully or in part, i.e. for one of multiple eligible comparisons; as is noted above for KQ3). In this case, we will contextualize and summarize the available evidence and perform certainty of evidence appraisals (based on information reported in the review) as needed to facilitate interpretation by the Task Force during guideline development. For the integration approach (detailed in Supplementary File 3), we will identify relevant studies in multiple previously published systematic reviews and develop and run update searches to present to identify contemporary studies not included in earlier reviews. The existing reviews will be used primarily to locate primary studies, although we may rely on reporting by reviews for some data extraction or risk of bias assessments, and will re-analyze the data using the primary studies and assess the overall certainty of the evidence in all cases. To identify potential candidate reviews, we undertook a comprehensive search for relevant systematic reviews, published between 2014 and March 2019, and scrutinized each for suitability. Important considerations included the comprehensiveness of the original searches, the scope of the review (i.e. ability to capture the studies of interest), and the reporting quality. Details of the reviews that we will use as a source of studies are in Supplementary File 4.

### Literature searches

We developed all database searches in collaboration with our research librarian. The searches, available in Supplementary File 5 for KQs 1, 2, and 4, have been peer reviewed by an external librarian according to PRESS (Peer Review of Electronic Search Strategies) guidance [105]. The searches for KQ 5 will be updated from previous reviews [63, 106, 107], with adaptations as needed. Unless otherwise indicated, all searches will be limited to studies published in English or French. We will not apply geographic filters to any of the searches. For KQ 1, we will contact five content experts by e-mail to inquire about their knowledge of additional relevant studies. We will contact each expert twice, 2 weeks apart, before ceasing contact if we do not receive a reply. In all cases, we will also search the reference lists of the included studies and of relevant systematic reviews identified during screening for additional records. We will search ClinicalTrials.gov and the World Health Organization International Clinical Trials Registry Platform for ongoing trials. Although we will exclude studies available only as conference proceedings, letters, or abstracts, we will contact the corresponding authors twice, 2 weeks apart, to ask about relevant full reports before ceasing contact if we do not receive a reply. The following are details of the strategies specific to each KQ. The results of the electronic database searches for all KQs will ultimately be combined into a single database (removing duplicates) to create efficiencies in screening (due to inevitable overlap across the searches).

For KQ 1, we will search Ovid Medline (1946-), Ovid Embase (1996-), and Cochrane Central (1996-) from 1995 onward using MeSH terms and keywords for cervical cancer and screening, and study design filters for RCTs and observational studies. We have chosen to develop and run de novo searches rather than updating the searches from the 2013 CTFPHC guideline review because that review did not include the incidence of CIN as an outcome, nor screening with hrHPV.

For KQ 2, we will integrate studies from the 2019 health technology assessment (HTA) on HPV testing for primary screening for the prevention and early detection of cervical cancer by the Canadian Agency for Drugs and Technologies in Health (CADTH) [63] and the 2018 systematic review by Arbyn et al. [106] on the comparative accuracy of self- vs. clinician-sampled hrHPV tests. We will update the searches for the CADTH review in Ovid Medline (1946-), Ovid Embase (1996-), and Cochrane Central (1996-) from 2016 onward to identify studies published after the last date searched (March 2017 for the full search), undertaking edits to the searches as necessary (e.g. removing concepts that are not relevant to our KQ 2). We will update the searches for the Arbyn et al. review in the same databases from 2017 onward (last date searched, April 2018). We anticipate the possibility that an update to the systematic review by Arbyn et al. may become available before we undertake our review for KQ 2. If such is the case, we will use the updated review as is without de novo searches or syntheses for the comparison of self- and clinician-sampled hrHPV testing.

CADTH sought to include systematic reviews and subsequently searched for primary studies published after the most recent systematic review. The inclusion of systematic reviews is not consistent with standard Task Force procedures for evidence synthesis [85]. Thus, we will supplement the updated database searches by screening the reference lists of the systematic reviews included in the CADTH HTA to identify the primary studies published prior to 2016.

For KQ 4, we will search Ovid Medline (1946-), Scopus (2004-), and EconLit (1886-) from 2000 onward using MeSH terms and keywords for cervical cancer, preferences and preference-based methods (e.g. conjoint analysis, trade-off), decision making, and attitudes.

For KQ 5, we will integrate studies (eligible for our review) from the 2011 Cochrane systematic review by Everett et al. on interventions to encourage cervical screening uptake [107] and the 2018 systematic review by Arbyn et al. on hrHPV self-sampling compared with reminders to encourage cervical screening rates [106]. The Cochrane review by Everett et al. included studies of interventions targeted at women to improve cervical screening rates, compared with no intervention or routine care [107]. We will update the Ovid Medline (1946-), Ovid Embase (1996-), and Cochrane Central (1996-) searches from 2008 onward to identify contemporary studies not included in the Cochrane review, undertaking edits to the searches as necessary. We expect the update search to capture studies of hrHPV self-sampling compared with reminders (as per Arbyn et al.’s review), and other effectiveness studies published since the last date searched in the review by Everett et al. As per KQ 2, we anticipate the possibility that an update to the systematic review by Arbyn et al. may become available before we undertake our review for KQ 5. If such is the case, we will use the updated review as is without de novo searches or syntheses for the comparison of self- and clinician-sampled hrHPV testing.

### Study selection

#### Electronic database searches

We will upload the results of the electronic searches to EndNote (v.X7, Clarivate Analytics, Philadelphia, PA) and remove duplicates. We will transfer the titles and abstracts to DistillerSR (Evidence Partners, Ottawa, Canada) for screening. Two reviewers will independently screen the studies for eligibility in two stages (titles and abstracts, then full texts) following the pre-defined selection criteria (Tables 2, 3, 4, and 5) and mark each as include/unsure or exclude. At the title and abstract screening stage, we will use the liberal-accelerated approach [108, 109], whereby any record marked as include/unsure by either of two reviewers will be considered eligible for full-text screening. Records excluded by either reviewer will be screened by a second reviewer to confirm or refute their exclusion. At the full-text screening stage, the reviewers will agree upon the included studies, with arbitration by a third reviewer if necessary. We will record the reasons for excluding full texts and illustrate the study selection process via a flow diagram. We will append a detailed list of the excluded studies, with full-text exclusion reasons, to the final report. Before each screening stage, we will undertake a pilot round of 200 titles and abstracts and 10 full texts, or as many as needed to achieve a mutual understanding of the selection criteria. To create efficiencies, we will screen for studies meeting the eligibility criteria for all KQs simultaneously (the searches for all KQs will ultimately be combined into one database).

When inadequate detail is reported in a study to confirm or refute its eligibility, we will contact the corresponding author by e-mail to request the additional information required. We will contact authors twice, 2 weeks apart, before ceasing contact if we do not receive a reply.

#### Studies identified via other sources

Studies identified via content experts and reference lists (i.e. of known systematic reviews that we are using as sources of studies, systematic reviews identified during screening, included studies) will be uploaded to separate folders in EndNote for storage and management. These will be screened following the same procedures as described for those identified via the electronic database searches. The selection process for these studies will be incorporated into the aforementioned flow diagram.

### Data extraction

For all KQs, we will develop standard forms in Excel v. 2016 (Microsoft Corporation, Redmond, WA) to guide data extraction. We will pilot test the forms on a random sample of 3 to 5 included studies for each KQ to ensure the complete and accurate extraction of all relevant data. Supplementary File 6 outlines the data extraction items for each KQ.

Data for the studies included in the review for each KQ will be extracted by one reviewer with verification by another, with the exception of results data (i.e. findings for the outcomes of interest) which will be independently extracted by two reviewers with consensus. A third reviewer will arbitrate if agreement on the extracted data cannot be reached. For qualitative studies (KQ 4), one reviewer will copy the relevant ‘Results’ or ‘Findings’ texts and paste them into a Word (Microsoft Corporation, Redmond, WA) document for analysis [110]. A second reviewer will verify the completeness of the extraction.

To create efficiencies, we will rely on the study characteristics and results data for primary studies reported in earlier systematic reviews, where feasible. In the case of reviews with high-quality conduct and reporting (e.g. Cochrane systematic reviews), one reviewer will perform a quality check of 10% of the data specific to the outcomes of interest, and unless substantial errors or omissions are noted, we will rely on the reported data without further re-extraction from the primary study. When the data of interest are incompletely reported, one reviewer will extract data from the primary study and compare data specific to the outcomes of interest (as previously described) to that reported in the earlier review(s) for consistency. A second reviewer will provide input only in cases where discrepancies between the extracted data and that reported in reports of earlier systematic reviews cannot be resolved.

Specific to KQ 2, we expect heterogeneity in the criteria (thresholds) used to define a positive test result across studies. Differences in the criteria for test-positivity across studies could affect whether and how we pool and interpret their results. We are not able to judge a priori the possible array of reported definitions. Thus, to inform our analyses, we will extract the definition of a positive test reported in the individual studies and present the range of definitions (without further study details) to clinical experts supporting the working group. Based on clinical expert judgment and improved familiarity with the range of definitions reported across studies, we will finalize our data analysis plan (i.e. which types of studies we may be able to pool). Only after the clinical experts have deliberated on the consistency and compatibility of available definitions and we have developed a suitable analysis plan will we move forward with the extraction of results data. Because we will finalize the analysis details prior to the extraction of data, and based on the input of clinicians who will not be aware of study details, the risk of biasing the analyses will be minimal.

### Risk of bias assessment

Considering the array of available risk of bias tools [111–113], we will use study design–specific tools that we believe best account for potential sources of bias [102, 114–118]. The planned methods are described in detail in Supplemental File 3. For all KQs, we will develop standard forms in Excel to guide risk of bias appraisal. We will pilot test the forms on 3 to 5 included studies for each KQ to ensure a mutual understanding of the requirements of each tool. We will report domain-specific risk of bias ratings for each included study, with justification for each rating, in an appendix to the final report. Two reviewers will independently appraise the risk of bias of each included study and reach consensus. A third reviewer will be consulted if an agreement cannot be reached. We will extract and use risk of bias appraisals reported in available systematic reviews where possible, to create efficiencies.

### Data synthesis

#### Key question 1: effectiveness and comparative effectiveness

Where appropriate, we will pool studies reporting on mortality from cervical cancer, all-cause mortality, the incidence of ICC, the incidence of CIN 2 and CIN 3, the number and rate of colposcopy and/or biopsy, and/or adverse pregnancy outcomes, per outcome-comparison. The measure of effect will be the relative risk (RR) or odds ratio (OR) with 95% confidence intervals (CIs), where appropriate. These will be calculated in Review Manager version 5.3 (The Nordic Cochrane Centre, The Cochrane Collaboration, Copenhagen, Denmark) from raw data reported in the studies or, if not provided, we will use the reported relative measures. When available, we will use adjusted ORs from observational studies, as these usually reduce the impact of confounding [119]. We will pool data using DerSimonian and Laird random effects models [120] to account for expected clinical and methodological heterogeneity across studies [121]. For rare events, we will use the Peto one-step odds ratio method to provide a less biased effect estimate [122], unless control groups are of unequal sizes, a large magnitude of effect is observed, or when events become more frequent (5 to 10%). In these cases, the reciprocal of the opposite treatment arm size correction will be used [122]. We will pool data from RCTs and controlled clinical trials separately from observational studies. We will present separate analyses for each comparison. In some cases, we may also deem it appropriate to combine intervention groups (e.g. for the comparisons of any screening vs. no screening) using standard methods to avoid unit of analysis issues [119]. We will transform the pooled RR for each outcome to the absolute risk reduction (ARR) via standard methods [123]. We will calculate the number needed to screen for an additional beneficial outcome for outcomes with statistically significant results.

We will consider false positives to be cervical screening tests that are positive (according to the primary testing strategy used in the individual studies, recognizing that definitions of test positivity will differ across studies) and lead to diagnostic follow-up testing, but that are not histologically confirmed as CIN 2, CIN 3, or more severe disease. We will calculate the false-positive rate using available data in the individual studies, as follows: (no. of individuals with a positive screening test result who are not histologically diagnosed with the relevant condition/no. of individuals not diagnosed with the relevant condition, regardless of screening test result). This calculation necessitates histological examination for pre-cancerous lesions on all participants. Should this information not be available (in the published report and following attempts to contact the study authors), we will report the number of positive tests and the total number of tests, as reported by the authors. The range of false-positive rates across studies will be reported narratively and in tables, per test.

We are not aware of a standard formula for estimating overdiagnosis in the context of cervical screening. Thus, we expect studies reporting on overdiagnosis to be highly methodologically heterogeneous. For this reason, we will synthesize data on this outcome narratively and in tables, including the method (formula) used to derive each estimate.

#### Key question 2: comparative accuracy

We will populate 2 × 2 tables with the true positive (TP), false positive (FP), true negative (TN), and false negative (FN) for each screening test used in each study. If we identify more than three studies that we deem suitable for statistical pooling, we will compare accuracy data per outcome and per comparison using the Rutter and Gatsonis hierarchical summary receiver operating characteristic (HSROC) model [124], as recommended by Cochrane [125]. This model allows for the exploration of heterogeneity in test positivity (threshold for a positive test), position of the HSROC curve (accuracy of the test), and the shape of the HSROC curve [125]. Compared to the binomial regression model, the HSROC model also more fully accounts for within- and between-study variability in TP and FP rates [124]. We will investigate whether test strategies are associated with the shape and position of the summary ROC curve by fitting a binary covariate to the model representing the type of test that informed each 2 × 2 table [125]. In the event that preliminary plots of the study level estimates of sensitivity and specificity in ROC space reveal substantial differences in heterogeneity between studies for the two tests being investigated, we will assess whether the assumption of equal variances of the random effects of the two tests is reasonable by comparing the fit of the alternative models (i.e. where variances do or do not depend on the covariate for test strategy) [125]. For each screening strategy, we will report the pooled relative sensitivity and specificity across studies, with 95% CIs. In the event that the data are not suitable for statistical pooling, we will report their findings narratively and in tables.

#### Key question 4: relative importance of potential outcomes from screening

We will synthesize the quantitative data separately from the qualitative data. For the quantitative data, we expect to undertake a narrative synthesis given the likely heterogeneity in study designs, exposures, comparisons, and outcomes reported across studies. We will synthesize the included studies and draw conclusions based on the body of evidence using standard methods for narrative syntheses, as described by Popay et al. [126]. Adaptations to standard methodology may be necessary, as our review aims to investigate peoples’ values and preferences, so the outcomes differ, to a certain extent, when compared with intervention or implementation reviews. We will first present an overall synthesis of each included study, including their characteristics and reported findings. We will then describe relationships within and between studies, focusing on our exposure subgroups and comparators of interest and other factors such as methodological quality. As much as possible, we intend to report a best estimate of values and preferences for various exposures and potential moderating factors.

We will analyze the qualitative data following standard procedures for thematic analysis [110, 127]. One reviewer will initially read through the data to familiarize themselves with the prevailing ideas. Next, the reviewer will use line-by-line coding in Microsoft Word to apply one or more codes to each line of text. The reviewer will then compare codes across the data, combine similar codes, categorize common codes into themes, and develop memos for each theme. To reduce the risk of interpretive biases, a second reviewer will review the codes and themes for differences in interpretation. The two reviewers will agree upon the final themes, with the input of a third reviewer if necessary. We will report on each theme narratively.

#### Key question 5: effectiveness and comparative effectives of interventions to increase screening rates

We will incorporate newly identified studies into the analyses previously reported in the Cochrane systematic review by Everett et al. [107]. Additional studies extracted from the review by Arbyn et al. [106] will be pooled via the same methods. In some cases, we may also deem it appropriate to combine intervention groups from multi-arm trials using standard methods to avoid unit of analysis issues [119]. We will transform the pooled RR for each outcome to the absolute values via standard methods [123]. We will calculate the number needed to treat for an additional beneficial outcome (i.e. participation) for outcomes with statistically significant results. We will report on studies that are not appropriate for statistical pooling narratively.

#### Dealing with missing data

When data required for statistical pooling are not reported by the individual studies, we will contact the corresponding author via e-mail to inquire about the availability of the data. We will contact authors twice, 2 weeks apart, before ceasing contact if we do not receive a response.

For randomized trials, we anticipate that many will report their findings based on a ‘number of individuals screened’ denominator, rather than intention-to-screen calculations using all individuals randomized. Our primary analysis will use outcome data derived by analyzing all individuals randomized (i.e. intention-to-screen). We will extract data as reported in the individual studies using the number randomized as the denominator for each arm. We will also analyze based on the findings as reported in the individual studies, undertaking separate analyses for studies reporting only the number of individuals screened and those reporting on all individuals randomized.

#### Unit of analysis issues

In the event of the inclusion of cluster-randomized trials, we will take appropriate measures to avoid unit-of-analysis errors when reporting their findings and/or incorporating them into meta-analysis [128]. When available, we will use the intracluster correlation coefficient (ICC) reported in the trial to apply a design effect to the sample size and number of events in each of the treatment and control groups [129]. If not reported, we will use an external estimate from similar studies. We will clearly identify cluster-randomized trial data when it is included in meta-analysis with individually randomized trials. Decisions about whether it is reasonable to pool data from cluster-randomized and individually randomized trials will be undertaken on a case-by-case basis. We will investigate the robustness of the conclusions from any meta-analysis including cluster-randomized trials via sensitivity analysis.

#### Assessment of heterogeneity

We will explore heterogeneity via subgroup analyses. First, we will report within-study subgroup data from our pre-specified subgroups of interest (Tables 2, 3, 4, and 5). We will also stratify the meta-analyses by subgroups (between-study analysis) or use other relevant statistical techniques like meta-regression to investigate heterogeneity. For population subgroups, we will use a large majority (i.e. > 80% of participants) to decide the relevant subgroup for each study. We will interpret the plausibility of subgroup differences cautiously using available guidance [130, 131]. Should within- or between-study subgroup analysis not be available or possible for some subgroups, studies with individuals or populations that may require equity (e.g. Indigenous peoples, trauma affected, low income) or other considerations by the Task Force will be noted and the applicability of the interventions to these populations will be assessed.

#### Small study bias

When meta-analyses of trials contain at least eight studies of varying sizes, we will test for small study bias visually by inspecting funnel plots for asymmetry and statistically via the Egger test [132].

### Certainty in the body of evidence

We will use GRADE methods [133] to assess the certainty of evidence for all outcomes, without relying on the appraisals reported in earlier systematic reviews. In the event that we use one or multiple systematic reviews as is to answer a KQ (e.g. the Kyrgiou et al. [100, 101] reviews for KQ 3), we will review the reported certainty of evidence appraisals and undertake amendments as necessary to ensure that the appraisals are appropriately contextualized. In cases where studies of interventions cannot be pooled in meta-analysis, we will use GRADE guidance for rating the certainty of evidence in the absence of a single estimate of effect [134]. Two reviewers will independently assess the certainty of evidence for each outcome and agree on the final assessments. A third reviewer will arbitrate if necessary.

We will assess the certainty of evidence (very low, low, moderate, or high) based on five considerations: study limitations (risk of bias), inconsistency of results, indirectness of evidence, imprecision, and publication (small study) bias [135–140]. We will assess the certainty of evidence from trials and observational studies separately, for each outcome. For KQs of intervention effects (KQs 1 and 5), data from RCTs will begin at high certainty and be downgraded for flaws in each of the aforementioned domains (or, rarely, upgraded for strengths) [141], whereas observational studies will begin at low certainty. For KQ 2 on diagnostic accuracy, all studies will begin at high certainty [142, 143]. For KQ 4, we will adhere to GRADE methods for assessing the certainty of evidence in the importance of outcomes or values and preferences [103, 117]. We will report our appraisals comprehensively and transparently, including justification for downgrading on any of the considered domains. We will use a partially contextualized approach; thus, we will express our certainty that the true estimate lies within a range of magnitudes for each outcome. We will not account for other outcomes when assessing the magnitude of effect for individual outcomes, nor consider the certainty of any one outcome vs. another [144].

For each KQ, we will create a separate GRADE summary of findings table [136]. Justifications for rating up or down in any of the considered domains will be explained. We will also note where differences were observed between the data from trials and that from observational studies, or when we have relied solely on either the trial or observational evidence. The certainty of evidence assessments for each outcome will be incorporated into the Task Force’s evidence-to-decision framework [145]. The Task Force may choose to fully contextualize the range of possible effects on all outcomes (including benefits and harms). The Task Force will consider the net benefits and harms of screening and other elements (e.g. costs, feasibility, patient values and preferences) to develop updated recommendations for screening for the prevention of cervical cancer [145].

### Task Force involvement

The Task Force and clinical experts will not be involved in the selection of studies, extraction of data, appraisal of risk of bias (or methodological quality), nor synthesis of data, but will contribute to the interpretation of the findings and comment on the draft report. Clinical experts and/or Task Force members may be called upon to contribute to the certainty of evidence appraisals, e.g. to interpret directness (applicability) of included studies to the population of interest for the recommendation.

## Discussion

Since the publication of the 2013 Task Force guideline for screening for the prevention and early detection of cervical cancer, new studies have become available that may alter recommendations. The proposed systematic reviews will identify and synthesize newly available studies, which will inform an update of the guideline. We anticipate some challenges to integrating studies reported in earlier systematic reviews. To mitigate potential challenges, we have planned methods (e.g. searching references lists, contacting experts, independent data extraction and/or quality checks) consistent with the highest standards for evidence synthesis. We are confident that the planned methods will identify and provide a rigorous evaluation of all studies critical to the update of the guideline.

## Supplementary Information

**Additional file 1.** PRISMA-P Checklist.

**Additional file 2.** Outcome Definitions and Baseline Risks.

**Additional file 3.** Methodological Details.

**Additional file 4.** Synopsis of Reviews Used as Sources of Studies of Study-level Data.

**Additional file 5.** Search Strategies for Key Questions 1, 2 and 4.

**Additional file 6.** Data Extraction Items for Each Key Question.

## Data Availability

Not applicable.

## Abbreviations

AGC: Atypical glandular cells
AIS: Adenocarcinoma in situ
ARR: Absolute risk reduction
ASCUS: Atypical squamous cells of undetermined significance
CADTH: Canadian Agency for Drugs and Technologies in Health
CI: Confidence interval
CIN: Cervical intraepithelial neoplasia
CTFPHC: Canadian Task Force on Preventive Health Care
DNA: Deoxyribonucleic acid
ERSC: Evidence Review and Synthesis Center
FN: False negative
FP: False positive
GRADE: Grading of Recommendations, Assessment, Development, and Evaluation
HPV: Human papillomavirus
hrHPV: High-risk human papillomavirus
HSIL: High-grade squamous epithelial lesion
HSROC: Hierarchical summary receiver operating characteristic
ICC: Invasive cervical cancer
KQ: Key question
LEEP: Loop electrosurgical excision procedure
LSIL: Low-grade squamous epithelial lesion
MeSH: Medical Subject Headings
PICOTS: Population, intervention/exposure, comparator(s), outcome(s), timeframe, study design, setting
PRESS: Peer Review of Electronic Search Strategies
PRISMA: Preferred Reporting Items for Systematic Reviews and Meta-analyses
RCT: Randomized controlled trial
RNA: Ribonucleic acid
RR: Risk ratio
SIL: Squamous epithelial lesion
TN: True negative
TP: True positive
USPSTF: United States Preventive Services Task Force

## References

[1] Stumbar SE, Stevens M, Feld Z (2019). Cervical cancer and its precursors: a preventative approach to screening, diagnosis, and management. Prim Care.

[2] Canadian Cancer Society (2019). Cervical cancer.

[3] Walboomers JM, Jacobs MV, Manos MM, Bosch FX, Kummer JA, Shah KV (1999). Human papillomavirus is a necessary cause of invasive cervical cancer worldwide. J Pathol.

[4] Volesky KD, El-Zein M, Franco EL, Brenner DR, Friedenreich CM, Ruan Y (2019). Cancers attributable to infections in Canada. Prev Med.

[5] Herrero R, Thun MJ, Linet MS, Cerhan JR, Haiman CA, Schottenfeld D (2018). Cervical cancer. Cancer epidemiology and prevention.

[6] Canadian Cancer Society (2019). HPV and cancer.

[7] Tricco AC, Ng CH, Gilca V, Anonychuk A, Pham B, Berliner S (2011). Canadian oncogenic human papillomavirus cervical infection prevalence: systematic review and meta-analysis. BMC Infect Dis.

[8] Chesson HW, Dunne EF, Hariri S, Markowitz LE (2014). The estimated lifetime probability of acquiring human papillomavirus in the United States. Sex Transm Dis.

[9] World Health Organization (2019). Human papillomavirus (HPV) and cervical cancer.

[10] Ramirez PT, Salvo G (2019). Cervical cancer: Merck manual.

[11] Schiffman M, Kjaer SK (2003). Chapter 2: natural history of anogenital human papillomavirus infection and neoplasia. J Natl Cancer Inst Monogr.

[12] Schiffman M, Clifford G, Buonaguro FM (2009). Classification of weakly carcinogenic human papillomavirus types: addressing the limits of epidemiology at the borderline. Infect Agent Cancer.

[13] Coutlée F, Ratnam S, Ramanakumar AV, Insinga RR, Bentley J, Escott N (2011). Distribution of human papillomavirus genotypes in cervical intraepithelial neoplasia and invasive cervical cancer in Canada. J Med Virol.

[14] Jaisamrarn U, Castellsagué X, Garland SM, Naud P, Palmroth J, Del Rosario-Raymundo MR (2013). Natural history of progression of HPV infection to cervical lesion or clearance: analysis of the control arm of the large, randomised PATRICIA study. PLoS One.

[15] Bernard E, Pons-Salort M, Favre M, Heard I, Delarocque-Astagneau E, Guillemot D (2013). Comparing human papillomavirus prevalences in women with normal cytology or invasive cervical cancer to rank genotypes according to their oncogenic potential: a meta-analysis of observational studies. BMC Infect Dis.

[16] Tainio K, Athanasiou A, Tikkinen KAO, Aaltonen R, Cárdenas J, Glazer-Livson S (2018). Clinical course of untreated cervical intraepithelial neoplasia grade 2 under active surveillance: systematic review and meta-analysis. BMJ..

[17] McCredie MR, Sharples KJ, Paul C, Baranyai J, Medley G, Jones RW (2008). Natural history of cervical neoplasia and risk of invasive cancer in women with cervical intraepithelial neoplasia 3: a retrospective cohort study. Lancet Oncol.

[18] McIndoe WA, McLean MR, Jones RW, Mullins PRJOG (1984). The invasive potential of carcinoma in situ of the cervix. Obstet Gynecol.

[19] Hildesheim A, Hadjimichael O, Schwartx PE, Wheeler CM, Barnes W, Lowell DM (1999). Risk factors for rapid-onset cervical cancer. Am J Obstet Gynecol.

[20] Schiffman M, Castle PE, Jeronimo J, Rodriguez AC, Wacholder S (2007). Human papillomavirus and cervical cancer. Lancet..

[21] Fu T-CJ, Carter JJ, Hughes JP, Feng Q, Hawes SE, Schwartz SM (2016). Re-detection vs. new acquisition of high-risk human papillomavirus in mid-adult women. Int J Cancer.

[22] Shew ML, Ermel AC, Tong Y, Tu W, Qadadri B, Brown DR (2015). Episodic detection of human papillomavirus within a longitudinal cohort of young women. J Med Virol.

[23] Liu S-H, Cummings DAT, Zenilman JM, Gravitt PE, Brotman RM (2014). Characterizing the temporal dynamics of human papillomavirus DNA detectability using short-interval sampling. Cancer Epidemiol Biomark Prev.

[24] Carter JJ, Koutsky LA, Hughes JP, Lee SK, Kuypers J, Kiviat N (2000). Comparison of human papillomavirus types 16, 18, and 6 capsid antibody responses following incident infection. J Infect Dis.

[25] Gravitt PE (2012). Evidence and impact of human papillomavirus latency. Open Virol J.

[26] Public Health England (2016). NHS cervical screening programme. Colposcopy and Programme Management.

[27] National Cancer Institute (2020). NCI dictionary of cancer terms.

[28] Peirson L, Fitzpatrick-Lewis D, Ciliska D, Warren R (2013). Screening for cervical cancer: a systematic review and meta-analysis. Syst Rev.

[29] Smith JHF, Patnik J (2013). Achievable standards, benchmarks for reporting, and criteria for evaluating cervical cytopathology.

[30] Nayar R, Wilbur DC (2015). The pap test and Bethesda 2014. Acta Cytol.

[31] Darragh TM, Colgan TJ, Cox JT, Heller DS, Henry MR, Luff RD (2013). The Lower Anogenital Squamous Terminology Standardization project for HPV-associated lesions: background and consensus recommendations from the College of American Pathologists and the American Society for Colposcopy and Cervical Pathology. Int J Gynecol Pathol.

[32] Sellors JW, Sankaranarayanan R (2003). Colposcopy and treatment of cervical intraepithelial neoplasia: a beginner’s manual.

[33] Brenner DR, Weir HK, Demers AA, Ellison LF, Louzado C, Shaw A (2020). Projected estimates of cancer in Canada in 2020. CMAJ..

[34] The International Collaboration of Epidemiological Studies of Cervical, C., Comparison of risk factors for invasive squamous cell carcinoma and adenocarcinoma of the cervix: collaborative reanalysis of individual data on 8,097 women with squamous cell carcinoma and 1,374 women with adenocarcinoma from 12 epidemiological studies. Int J Cancer. 2007;120(4):885-91. 10.1002/ijc.22357 17131323

[35] Navaneelan T (2015). Trends in the incidence and mortality of female reproductive system cancers. Health at a Glance. Statistics Canada Catologue no 82-624-X.

[36] Popadiuk C, Stankiewicz A, Dickinson J, Pogany L, Miller AB, Onysko J (2012). Invasive cervical cancer incidence and mortality among canadian women aged 15 to 29 and the impact of screening. J Obstet Gynaecol Can.

[37] Canadian Cancer Statistics Advisory Committee (2018). Canadian cancer statistics 2018.

[38] Benard VB, Watson M, Saraiya M, Harewood R, Townsend JS, Stroup AM (2017). Cervical cancer survival in the United States by race and stage (2001-2009): findings from the CONCORD-2 study. Cancer..

[39] Markowitz LE, Tsu V, Deeks SL, Cubie H, Wang SA, Vicari AS (2012). Human papillomavirus vaccine introduction—the first five years. Vaccine..

[40] Public Health Agency of Canada (2018). Canadian immunization guide: part 4—active vaccines.

[41] Kreimer AR, Rolando H, Sampson JN, Porras C, Lowy DR, Schiller JT (2018). Evidence for single-dose protection by the bivalent HPV vaccine—review of the Costa Rica HPV vaccine trial and future research studies. Vaccine.

[42] Arbyn M, Xu L, Simoens C, Martin-Hirsch PPL (2018). Prophylactic vaccination against human papillomaviruses to prevent cervical cancer and its precursors. Cochrane Database Syst Rev.

[43] Bergman H, Buckley BS, Villanueva G, Petkovic J, Garritty C, Lutje V (2019). Comparison of different human papillomavirus (HPV) vaccine types and dose schedules for prevention of HPV-related disease in females and males. Cochrane Database Syst Rev.

[44] Canadian Partnership Against Cancer (2018). Cervical cancer screening in Canada: environmental scan.

[45] Markowitz LE, Liu G, Hariri S, Steinau M, Dunne EF, Unger ER (2016). Prevalence of HPV after introduction of the vaccination program in the United States. Pediatrics..

[46] Steben M, Tan Thompson M, Rodier C, Mallette N, Racovitan V, DeAngelis F (2018). A review of the impact and effectiveness of the quadrivalent human papillomavirus vaccine: 10 years of clinical experience in Canada. J Obstet Gynaecol Can.

[47] Kitchener H (2019). Optimising future cervical screening strategies. Papillomavirus Res.

[48] Volesky KD, El-Zein M, Franco EL, Brenner DR, Friedenreich CM, Ruan Y (2019). Estimates of the future burden of cancer attributable to infections in Canada. Prev Med.

[49] El-Zein M, Richardson L, Franco EL (2016). Cervical cancer screening of HPV vaccinated populations: cytology, molecular testing, both or none. J Clin Virol.

[50] Maxim LD, Niebo R, Utell MJ (2014). Screening tests: a review with examples. Inhal Toxicol.

[51] Landy R, Windridge P, Gillman MS, Sasieni PD (2018). What cervical screening is appropriate for women who have been vaccinated against high risk HPV? A simulation study. Int J Cancer.

[52] Shaw PA. The history of cervical screening I: the pap. test. 2000;22(2):110–4.

[53] Koliopoulos G, Nyaga VN, Santesso N, Bryant A, Martin-Hirsch PPL, Mustafa RA (2017). Cytology versus HPV testing for cervical cancer screening in the general population. Cochrane Database Syst Rev.

[54] Ogilvie GS, van Niekerk D, Krajden M, Smith LW, Cook D, Gondara L (2018). Effect of screening with primary cervical HPV testing vs cytology testing on high-grade cervical intraepithelial neoplasia at 48 months: the HPV FOCAL randomized clinical trial. JAMA..

[55] Ronco G, Giorgi-Rossi P, Carozzi F, Confortini M, Dalla Palma P, Del Mistro A (2010). Efficacy of human papillomavirus testing for the detection of invasive cervical cancers and cervical intraepithelial neoplasia: a randomised controlled trial. Lancet Oncol.

[56] Leinonen MK, Nieminen P, Lonnberg S, Malila N, Hakama M, Pokhrel A (2012). Detection rates of precancerous and cancerous cervical lesions within one screening round of primary human papillomavirus DNA testing: prospective randomised trial in Finland. BMJ..

[57] Canfell K, Caruana M, Gebski V, Darlington-Brown J, Heley S, Brotherton J (2017). Cervical screening with primary HPV testing or cytology in a population of women in which those aged 33 years or younger had previously been offered HPV vaccination: results of the Compass pilot randomised trial. PLoS Med.

[58] Ronco G, Giorgi-Rossi P, Carozzi F, Dalla Palma P, Del Mistro A, De Marco L (2006). Human papillomavirus testing and liquid-based cytology in primary screening of women younger than 35 years: results at recruitment for a randomised controlled trial. Lancet Oncol.

[59] Rijkaart DC, Berkhof J, Rozendaal L, van Kemenade FJ, Bulkmans NW, Heideman DA (2012). Human papillomavirus testing for the detection of high-grade cervical intraepithelial neoplasia and cancer: final results of the POBASCAM randomised controlled trial. Lancet Oncol.

[60] Naucler P, Ryd W, Tornberg S, Strand A, Wadell G, Elfgren K (2007). Human papillomavirus and Papanicolaou tests to screen for cervical cancer. N Engl J Med.

[61] Kitchener HC, Almonte M, Thomson C, Wheeler P, Sargent A, Stoykova B (2009). HPV testing in combination with liquid-based cytology in primary cervical screening (ARTISTIC): a randomised controlled trial. Lancet Oncol.

[62] Poli UR, Gowrishankar S, Swain M, Jeronimo J (2018). Triage of women testing positive with the careHPV test on self-collected vaginal samples for cervical cancer screening in a low-resource setting. J Glob Oncol.

[63] Chao YS, Clark M, Carson E, Weeks L, Moulton K, McFaul S, McLauchlin CM (2019). HPV Testing for primary cervical cancer screening: a health technology assessment.

[64] Pathak N, Dodds J, Zamora J, Khan K (2014). Accuracy of urinary human papillomavirus testing for presence of cervical HPV: systematic review and meta-analysis. BMJ..

[65] Cook DA, Smith LW, Law JH, Mei W, Gondara L, van Niekerk DJ (2018). Comparative performance of human papillomavirus messenger RNA versus DNA screening tests at baseline and 48 months in the HPV FOCAL trial. J Clin Virol.

[66] Iftner T, Becker S, Neis K-J, Castanon A, Iftner A, Holz B (2015). Head-to-head comparison of the RNA-based aptima human papillomavirus (HPV) assay and the DNA-based hybrid capture 2 HPV test in a routine screening population of women aged 30 to 60 years in Germany. J Clin Microbiol.

[67] Melnikow J, Henderson JT, Burda BU, Senger CA, Durbin S, Weyrich MS (2018). Screening for cervical cancer with high-risk human papillomavirus testing: updated evidence report and systematic review for the US Preventive Services Task Force. JAMA..

[68] Hakama M, Chamberlain J, Day NE, Miller AB, Prorok PC (1985). Evaluation of screening programmes for gynaecological cancer. Br J Cancer.

[69] Hakama M, Franco E, Monsonego J (1997). Screening for cervical cancer: experience of the Nordic countries. New developments in cervical cancer screening and prevention.

[70] Canadian Partnership Against Cancer (2018). Cervical cancer screening in Canada: environmental scan.

[71] Zhang Y, Coello PA, Brozek J, Wiercioch W, Etxeandia-Ikobaltzeta I, Akl EA (2017). Using patient values and preferences to inform the importance of health outcomes in practice guideline development following the GRADE approach. Health Qual Life Outcomes.

[72] Curry SJ, Krist AH, Owens DK, Barry MJ, Caughey AB, Davidson KW (2018). Screening for cervical cancer: US Preventive Services Task Force recommendation statement. JAMA..

[73] Statistics Canada (2018). Health fact sheets: cancer screening, 2017.

[74] Ahmed S, Shahid RK, Episkenew JA (2015). Disparity in cancer prevention and screening in aboriginal populations: recommendations for action. Curr Oncol.

[75] Elit L, Krzyzanowska M, Saskin R, Barbera L, Razzaq A, Lofters A (2012). Sociodemographic factors associated with cervical cancer screening and follow-up of abnormal results. Can Fam Physician.

[76] Farley M, Golding JM, Minkoff JR (2002). Is a history of trauma associated with a reduced likelihood of cervical cancer screening?. J Fam Pract.

[77] Kouyoumdjian FG, McConnon A, Herrington ERS, Fung K, Lofters A, Hwang SW (2018). Cervical cancer screening access for women who experience imprisonment in Ontario, Canada. JAMA Netw Open.

[78] Kerner J, Liu J, Wang K, Fung S, Landry C, Lockwood G (2015). Canadian cancer screening disparities: a recent historical perspective. Curr Oncol.

[79] Committee on Health Care for Underserved Women (2011). Committee Opinion no. 512: health care for transgender individuals. Obstet Gynecol.

[80] Canadian Task Force on Preventive Health C (2013). Recommendations on screening for cervical cancer. CMAJ..

[81] Health PEI (2020). Cervical cancer screening. Staff Resource Centre.

[82] Cancer Council Australia Cervical Cancer Screening Guidelines Working Party (2016). National Cervical Screening Program: guidelines for the management of screen-detected abnormalities, screening in specific populations and investigation of abnormal vaginal bleeding.

[83] United Kingdom National Screening Committee (2016). The UK NSC recommendation on cervical cancer screening in women.

[84] van Ballegooijen M, Hermens R (2000). Cervical cancer screening in the Netherlands. Eur J Cancer.

[85] Canadian Task Force on Preventive Health Care (2014). Procedure manual: Canadian Task Force on Preventive Health Care.

[86] Moher D, Shamseer L, Clarke M, Ghersi D, Liberati A, Petticrew M (2015). Preferred reporting items for systematic review and meta-analysis protocols (PRISMA-P) 2015 statement. Syst Rev.

[87] Guyatt GH, Oxman AD, Kunz R, Atkins D, Brozek J, Vist G (2011). GRADE guidelines: 2. Framing the question and deciding on important outcomes. J Clin Epidemiol.

[88] Moher D, Liberati A, Tetzlaff J, Altman DG (2009). Preferred reporting items for systematic reviews and meta-analyses: the PRISMA statement. PLoS Med.

[89] Ronco G, Giorgi-Rossi P, Carozzi F, Confortini M, Dalla Palma P, Del Mistro A (2008). Results at recruitment from a randomized controlled trial comparing human papillomavirus testing alone with conventional cytology as the primary cervical cancer screening test. J Natl Cancer Inst.

[90] Ogilvie GS, van Niekerk DJ, Krajden M, Martin RE, Ehlen TG, Ceballos K (2010). A randomized controlled trial of human papillomavirus (HPV) testing for cervical cancer screening: trial design and preliminary results (HPV FOCAL Trial). BMC Cancer.

[91] Cook DA, Mei W, Smith LW, van Niekerk DJ, Ceballos K, Franco EL (2015). Comparison of the Roche cobas(R) 4800 and Digene Hybrid Capture(R) 2 HPV tests for primary cervical cancer screening in the HPV FOCAL trial. BMC Cancer.

[92] Ogilvie GS, Krajden M, van Niekerk D, Smith LW, Cook D, Ceballos K (2017). HPV for cervical cancer screening (HPV FOCAL): complete round 1 results of a randomized trial comparing HPV-based primary screening to liquid-based cytology for cervical cancer. Int J Cancer.

[93] Ronco G, Segnan N, Giorgi-Rossi P, Zappa M, Casadei GP, Carozzi F (2006). Human papillomavirus testing and liquid-based cytology: results at recruitment from the new technologies for cervical cancer randomized controlled trial. J Natl Cancer Inst.

[94] Bulkmans NW, Rozendaal L, Snijders PJ, Voorhorst FJ, Boeke AJ, Zandwijken GR (2004). POBASCAM, a population-based randomized controlled trial for implementation of high-risk HPV testing in cervical screening: design, methods and baseline data of 44,102 women. Int J Cancer.

[95] Dijkstra MG, van Zummeren M, Rozendaal L, van Kemenade FJ, Helmerhorst TJ, Snijders PJ (2016). Safety of extending screening intervals beyond five years in cervical screening programmes with testing for high risk human papillomavirus: 14 year follow-up of population based randomised cohort in the Netherlands. BMJ..

[96] Elfstrom KM, Smelov V, Johansson AL, Eklund C, Naucler P, Arnheim-Dahlstrom L (2014). Long term duration of protective effect for HPV negative women: follow-up of primary HPV screening randomised controlled trial. BMJ..

[97] Kitchener HC, Fletcher I, Roberts C, Wheeler P, Almonte M, Maguire P (2008). The psychosocial impact of human papillomavirus testing in primary cervical screening-a study within a randomized trial. Int J Gynecol Cancer.

[98] Kitchener HC, Almonte M, Gilham C, Dowie R, Stoykova B, Sargent A (2009). ARTISTIC: a randomised trial of human papillomavirus (HPV) testing in primary cervical screening. Health Technol Assess.

[99] Kitchener HC, Canfell K, Gilham C, Sargent A, Roberts C, Desai M (2014). The clinical effectiveness and cost-effectiveness of primary human papillomavirus cervical screening in England: extended follow-up of the ARTISTIC randomised trial cohort through three screening rounds. Health Technol Assess.

[100] Kyrgiou M, Mitra A, Arbyn M, Paraskevaidi M, Athanasiou A, Martin-Hirsch PPL (2015). Fertility and early pregnancy outcomes after conservative treatment for cervical intraepithelial neoplasia. Cochrane Database Syst Rev.

[101] Kyrgiou M, Athanasiou A, Kalliala IEJ, Paraskevaidi M, Mitra A, Martin-Hirsch PPL (2017). Obstetric outcomes after conservative treatment for cervical intraepithelial lesions and early invasive disease. Cochrane Database Syst Rev.

[102] Zhang Y, Alonso-Coello P, Guyatt GH, Yepes-Nunez JJ, Akl EA, Hazlewood G (2019). GRADE Guidelines: 19. Assessing the certainty of evidence in the importance of outcomes or values and preferences—risk of bias and indirectness. J Clin Epidemiol.

[103] Zhang Y, Coello PA, Guyatt GH, Yepes-Nunez JJ, Akl EA, Hazlewood G (2019). GRADE guidelines: 20. Assessing the certainty of evidence in the importance of outcomes or values and preferences-inconsistency, imprecision, and other domains. J Clin Epidemiol.

[104] Robinson KA, Chou R, Berkman ND, Newberry SJ, Fu R, Hartling L (2016). Twelve recommendations for integrating existing systematic reviews into new reviews: EPC guidance. J Clin Epidemiol.

[105] McGowan J, Sampson M, Salzwedel DM, Cogo E, Foerster V, Lefebvre C (2016). PRESS peer review of electronic search strategies: 2015 guideline statement. J Clin Epidemiol.

[106] Arbyn M, Smith SB, Temin S, Sultana F, Castle P (2018). Detecting cervical precancer and reaching underscreened women by using HPV testing on self samples: updated meta-analyses. BMJ..

[107] Everett T, Bryant A, Griffin MF, Martin-Hirsch PP, Forbes CA, Jepson RG (2011). Interventions targeted at women to encourage the uptake of cervical screening. Cochrane Database Syst Rev.

[108] Khangura S, Konnyu K, Cushman R, Grimshaw J, Moher D (2012). Evidence summaries: the evolution of a rapid review approach. Syst Rev.

[109] O’Blenis P (2017). One simple way to speed up your screening process.

[110] Thomas J, Harden A (2008). Methods for the thematic synthesis of qualitative research in systematic reviews. BMC Med Res Methodol.

[111] Page MJ, McKenzie JE, Higgins JPT (2018). Tools for assessing risk of reporting biases in studies and syntheses of studies: a systematic review. BMJ Open.

[112] Farrah K, Young K, Tunis MC, Zhao L (2019). Risk of bias tools in systematic reviews of health interventions: an analysis of PROSPERO-registered protocols. Syst Rev.

[113] Quigley JM, Thompson JC, Halfpenny NJ, Scott DA (2019). Critical appraisal of nonrandomized studies—a review of recommended and commonly used tools. J Eval Clin Pract.

[114] Higgins JPT, Altman DG, Gøtzsche PC, Jüni P, Moher D, Oxman AD (2011). The Cochrane Collaboration’s tool for assessing risk of bias in randomised trials. BMJ..

[115] Wells GA, Shea B, O'Connell D, Peterson J, Welch V, Losos M, Tugwell P (2019). The Newcastle-Ottawa Scale (NOS) for assessing the quality of nonrandomised studies in meta-analyses.

[116] Cochrane Effective Practice and Organisation of Care (2019). Suggested risk of bias criteria for EPOC reviews.

[117] Whiting PF, Rutjes AW, Westwood ME, Mallett S, Deeks JJ, Reitsma JB (2011). QUADAS-2: a revised tool for the quality assessment of diagnostic accuracy studies. Ann Intern Med.

[118] Critical Appraisals Skills Programme (2018). CASP Checklists.

[119] Higgins JPT, Tianjing L, Deeks JD, Higgins JPT, Tianjing L, Chandler J, Cumpston M, Li T, Page MJ, Welch VA (2019). Chapter 6: choosing effect measures and computing estimates of effect. Cochrane handbook for systematic reviews of interventions, version 6.0. Cochrane.

[120] DerSimonian R, Laird N (1986). Meta-analysis in clinical trials. Control Clin Trials.

[121] Deeks JJ, Higgins JPT, Altman DG, Higgins JPT, Thomas J, Chandler J, Cumpston M, Li T, Page MJ, Welch VA (2019). Chapter 10: analysing data and undertaking meta-analyses. Cochrane handbook for systematic reviews of interventions, version 6.0. Cochrane.

[122] Sweeting MJ, Sutton AJ, Lambert PC (2004). What to add to nothing? Use and avoidance of continuity corrections in meta-analysis of sparse data. Stat Med.

[123] Schunemann HJ, Higgins JPT, Vist GE, Glasziou P, Akl EA, Skoetz N, Guyatt GH, Higgins JPT, Thomas J, Chandler J, Cumpston M, Li T, Page MJ, Welch VA (2019). Chapter 14: Completing ‘summary of findings’ tables and grading the certainty of evidence. Cochrane handbook for systematic reviews of interventions, version 6.0. Cochrane.

[124] Rutter CM, Gatsonis CA (2001). A hierarchical regression approach to meta-analysis of diagnostic test accuracy evaluations. Stat Med.

[125] Macaskill P, Gatsonis C, Deeks JJ, Harbord RM, Takwoingi Y, Deeks JJ, Bossuyt PM, Gatsonis C (2010). Chapter 10: Analysing and presenting results. Cochrane handbook for systematic reviews of diagnostic test accuracy, version 1.0. The Cochrane Collaboration.

[126] Popay J, Roberts H, Sowden A, Petticrew M, Arai L, Rodgers M, et al. Guidance on the conduct of narrative synthesis in systematic reviews. A product from the ESRC Methods Programme. 2006;1:b92.

[127] Vaismoradi M, Turunen H, Bondas T (2013). Content analysis and thematic analysis: implications for conducting a qualitative descriptive study. Nurs Health Sci.

[128] Higgins JPT, Eldridge S, Li T, Higgins JPTTJ, Chandler J, Cumpston M, Li T, Page MJ, Welch VA (2019). Chapter 23: Including variants on randomized trials. Cochrane handbook for systematic reviews of interventions, version 6.0. Cochrane.

[129] Rao JN, Scott AJ (1992). A simple method for the analysis of clustered binary data. Biometrics..

[130] Richardson M, Garner P, Donegan S (2019). Interpretation of subgroup analyses in systematic reviews: a tutorial. Clin Epidemiol Glob Health.

[131] Oxman AD, Guyatt GH (1992). A consumer’s guide to subgroup analyses. Ann Intern Med.

[132] Egger M, Smith GD, Schneider M, Minder C (1997). Bias in meta-analysis detected by a simple, graphical test. BMJ..

[133] Schunemann H, Brozek J, Guyatt G, Oxman A. GRADE handbook for grading quality of evidence and strength of recommendations: The GRADE Working Group; 2013. https://gdt.gradepro.org/app/handbook/handbook.html. Accessed 5 Jun 2020.

[134] Murad MH, Mustafa RA, Schunemann HJ, Sultan S, Santesso N (2017). Rating the certainty in evidence in the absence of a single estimate of effect. Evid Based Med.

[135] Atkins D, Best D, Briss PA, Eccles M, Falck-Ytter Y, Flottorp S (2004). Grading quality of evidence and strength of recommendations. BMJ..

[136] Guyatt GH, Oxman AD, Akl EA, Kunz R, Vist G, Brozek J (2011). GRADE guidelines: 1. Introduction—GRADE evidence profiles and summary of findings tables. J Clin Epidemiol.

[137] Guyatt GH, Oxman AD, Montori V, Vist G, Kunz R, Brozek J (2011). GRADE guidelines: 5. Rating the quality of evidence—publication bias. J Clin Epidemiol.

[138] Guyatt GH, Oxman AD, Kunz R, Woodcock J, Brozek J, Helfand M (2011). GRADE guidelines: 7. Rating the quality of evidence—inconsistency. J Clin Epidemiol.

[139] Guyatt GH, Oxman AD, Kunz R, Woodcock J, Brozek J, Helfand M (2011). GRADE guidelines: 8. Rating the quality of evidence—indirectness. J Clin Epidemiol.

[140] Guyatt GH, Oxman AD, Kunz R, Brozek J, Alonso-Coello P, Rind D (2011). GRADE guidelines 6. Rating the quality of evidence—imprecision. J Clin Epidemiol.

[141] Balshem H, Helfand M, Schünemann HJ, Oxman AD, Kunz R, Brozek J (2011). GRADE guidelines: 3. Rating the quality of evidence. J Clin Epidemiol.

[142] Schünemann HJ, Mustafa RA, Brozek J, Steingart KR, Leeflang M, Murad MH (2020). GRADE guidelines: 21 part 1. Study design, risk of bias, and indirectness in rating the certainty across a body of evidence for test accuracy. J Clin Epidemiol.

[143] Schünemann HJ, Mustafa RA, Brozek J, Steingart KR, Leeflang M, Murad MH (2020). GRADE guidelines: 21 part 2. Test accuracy: inconsistency, imprecision, publication bias, and other domains for rating the certainty of evidence and presenting it in evidence profiles and summary of findings tables. J Clin Epidemiol.

[144] Hultcrantz M, Rind D, Akl EA, Treweek S, Mustafa RA, Iorio A (2017). The GRADE Working Group clarifies the construct of certainty of evidence. J Clin Epidemiol.

[145] Andrews J, Guyatt G, Oxman AD, Alderson P, Dahm P, Falck-Ytter Y (2013). GRADE guidelines: 14. Going from evidence to recommendations: the significance and presentation of recommendations. J Clin Epidemiol.

