# Supplementary material for: Screening for the prevention and early detection of cervical cancer: protocol for systematic reviews to inform Canadian recommendations
Source: Syst Rev. 2021 Jan 2;10:2. doi: 10.1186/s13643-020-01538-9 (PMC7777363; doi:10.1186/s13643-020-01538-9)
Supplement: Supplementary file 2 — Additional file 2. Outcome Definitions and Baseline Risks. [file 13643_2020_1538_MOESM2_ESM.docx]

**Additional File 2. Outcome Definitions and Baseline Risks**

| **Outcome** | **Definition** | **Baseline estimate (when available)** | **Importance (when available)** |
| --- | --- | --- | --- |
| Incidence of invasive cervical cancer (squamous and adenocarcinoma) | Cancer that has spread from the surface of the cervix to tissue deeper in the cervix or to other parts of the body.^1^  Squamous and adenocarcinoma are the most common types of cervical cancer. | In 2017, an estimated 1,550 Canadian women were diagnosed with cervical cancer,^2^ which translates into an age-standardized rate of newly diagnosed cases of 8.3/100,000 and a 1/152 lifetime probability of developing cervical cancer.^3^ | In 2017, cervical cancer was the 13th most diagnosed cancer among females in Canada.^2^ Between 2012 and 2014, the predicted 5-year survival rates for cervical cancer was 72% and the 10-year survival rate was 67%.^3^  Well-organized, high-quality screening programs (using Pap testing at 3- to 5-year intervals) have been shown to prevent up to 80% of cervical cancers and well-organized programs have been found to reduce mortality from cervical cancer by up to 80%.^4^  Studies have found a cervical cancer diagnosis significantly impacts a women's psychosexual and functional health-related quality of life (HRQoL). A systematic review of HRQoL found an invasive cancer diagnosis was associated with HRQoL valuations ranging from 0.152 to 0.58.^5^ |
| Incidence of cervical intraepithelial neoplasia (CIN) | Precancerous changes of the cervix, identified at varying levels of severity:^2^  CIN2: high-grade squamous cell epithelial lesion (HSIL) with diagnostic uncertainty (could be low grade)  CIN3: HSIL | In comparison with cytology screening, primary hrHPV screening results in higher CIN2+ and CIN3+ detection rates in the initial round of screening. For example, in the HPV FOCAL trial, at 12-month follow-up, the CIN3+ detection rate for HPV testing versus cytology was 7.5/1,000 and 4.6/1,000, respectively. For CIN2+ detection, the corresponding rates for HPV and cytology testing were 16.5/1,000 and 10.1/1,000.^6^ However, at 48-month follow-up, the detection of CIN3+ and CIN2+ was significantly lower in the HPV group compared to the cytology group.^7^ | In a historical cohort of women whose cervical lesions were managed conservatively, rates of regression of CIN2+ to normal during a second screening test was found to be 6.9% within 2 years, 29.0% within 5 years and 53.7% within 10 years.^8^  Of women with CIN3+ on biopsy that are untreated, about one-third will go on to have invasive cervical cancer or vaginal cancer within 30 years. About half have persistent disease at 24 months. Of those who receive appropriate treatment, less than 1% go on to have cancer.^9^ |
| Cervical cancer mortality | Death due to cervical cancer | In 2017, an estimated 400 Canadian women died from cervical cancer (3), which corresponds to an age-standardized rate of death of 2.0/100,000 and a 1/426 lifetime probability of dying from cervical cancer.^3^ |  |
| All-cause mortality | Death due to any cause, including cervical cancer | In 2016, the age-standardized rate of death in Canada was 737/100,000 and the total number of deaths was 267,213.^10^ | The list of critical and important outcomes must include mortality unless a compelling rationale is provided for not including this outcome. |
| Overdiagnosis of CIN2, CIN3, invasive cervical cancer | The identification of cervical abnormalities, either from a screening test or follow-up testing, that would never go on to cause harm, would not progress, would progress too slowly to cause symptoms or harm during a person's remaining lifetime, or that go away without any treatment.^11^ | This is only recently being investigated for cervical cancer. The frequency has been high in recent reports:^12^ overdiagnosis rate of 74.8% (including pre-invasive disease; 3,999/100,000).^13^ Higher rates are found for HPV testing compared to cytology.^14^ | Women who are overdiagnosed (although not known to them) may experience psychological (e.g., fear, uncertainty) and physical (e.g., sleep problems) harms from a diagnosis that will never harm them.^15^ They may undergo treatments which provide no benefits, only harms.^11^ |
| False-positive rate for detecting CIN2, CIN3, invasive cervical cancer | False positives occur when individuals have positive screening test results but do not have cervical abnormalities or cancer diagnosed upon further examination. |  | Women who are incorrectly diagnosed with a lesion or cancer may experience psychological (e.g., fear, uncertainty) and physical (e.g., sleep problems) harms. They may undergo unnecessary diagnostic procedures which provide no benefits, only harms. |
| Adverse pregnancy outcomes from conservative, local management of CIN:  a. Early pregnancy (<20 weeks): miscarriage, cervical incompetence, cerclage  b. Late pregnancy (≥20 weeks): pre-term birth, cerclage, low birth weight | Miscarriage: Loss of a pregnancy within the first 20 weeks.  Cervical incompetence: Presumed weakness of cervical tissue that contributes to or causes premature delivery not explained by another abnormality.^18^  Cerclage: The placing of a nonabsorbable suture around the opening of an incompetent cervix.^19^  Pre-term birth: a birth that occurs before the start of the 37th week of pregnancy.  Low birth weight: A fetus or newborn weighing <2,500 g,^20^ based upon a weight obtained immediately after birth.^21^ | Miscarriages are relatively common, occurring in 15-20% (around one-sixth) of all pregnancies.  In Canada, approximately 8% of live births are pre-term (between 32 to <37 weeks of pregnancy). The rate of very pre-term birth (<32 weeks of pregnancy) is 1.2 to 1.3%.  In Canada, 9.1% (9.0 to 9.2%) of infants born singly (as opposed to a multiple birth, like twins) are born small for the stage of the pregnancy (gestational age).^22^ | Miscarriage can result in intense emotional distress and, in 20% of women, depression and/or anxiety can persist for up to 3 years, negatively affecting overall quality of life.^20^  Cervical incompetence is a contributor to premature delivery. The condition requires surgical intervention (i.e., cerclage).  Low birth weight increases the risk of infant death. It can result in diseases or symptoms of disease that occur suddenly (e.g., respiratory distress syndrome, necrotizing enterocolitis [a part of the digestive system called the intestine dies], jaundice [a disease that causes yellowing of the skin and whites of the eyes]) or develop over longer periods of time (e.g., increased risk of chronic conditions like diabetes, high blood pressure, intellectual or developmental disability).^23^ |
| Number and rates of colposcopy and/or biopsy, including LEEP (or referral rate) | Number and proportion of women with a positive screening test result who received diagnosis based on a microscopic examination of cervical tissue within a 12 month period.^16^  Colposcopy is a medical procedure that uses a special instrument called a colposcope to closely examine the cervix, vagina and vulva [clitoris, vaginal lips and vaginal opening] for signs of disease. A colposcopy may be recommended if you have a positive cervical screening test.  LEEP stands for Loop Electrosurgical Excision Procedure and is a type of treatment that removes abnormal cells from the cervix to prevent cervical cancer. | In 2009-10, 82-97% of Canadian women who had a high-grade [an area of cells on the surface of the cervix that when examined under a microscope look very abnormal] or more severe Pap test [a procedure in which cells are removed from the cervix with a small brush or spatula so they can be examined under a microscope] result and colposcopy had a biopsy within in 12 months.^17^ | Timely colposcopy or biopsy following a positive screening result is a key component of effective cervical cancer prevention.^17^  As primary HPV screening has a higher detection rate of CIN2+ and CIN3+ on initial screening compared to cytology screening, colposcopy referral rates are also initially higher with primary HPV screening. For example, in the HPV FOCAL trial referral rates for colposcopy were 57.0/1,000 for the HPV screening group compared to 30.8/1,000 for the cytology group. However, at 48-month follow-up rates of colposcopy referrals were lower in the HPV screened group (49.2/1,000) compared to the cytology group (70.5/1,000). The cumulative colposcopy referral rates were similar between groups (HPV group: 106.2/1,000; cytology group: 101.5/1,000).^7^ |

**References**

1. National Cancer Institude. NCI dictionary of cancer terms. 2016. Available at: https://www.cancer.gov/widgets/termdictionarywidgetenglish. Accessed 13 September 2019.
2. Waxman AG, Chelmow D, Darragh TM, et al. Revised terminology for cervical histopathology and its implications for management of high-grade squamous intraepithelial lesions of the cervix. Obstet Gynecol. 2012;120(6):1465-71.
3. Canadian Cancer Statistics Advisory Committee. Canadian Cancer Statistics 2019. Toronto, Ontario: Canadian Cancer Society; 2019. Available at: https://www.cancer.ca/en/cancer-information/cancer-101/canadian-cancer-statistics-publication/?region=on. Accessed 13 September 2019.
4. International Agency for Research on Cancer (IARC). Cervix Cancer Screening: IARC Handbooks of Cancer Prevention, Vol. 10. Lyon: IARC, 2005.
5. Céilleachair O, O'Mahony JF, O'Connor M, et al. Health-related quality of life as measured by the EQ-5D in the prevention, screening and management of cervical disease: A systematic review. Qual Life Res. 2017;26:2885-97.
6. Ogilvie GS, Krajden M, van Niekerk D, et al. HPV for cervical cancer screening (HPV FOCAL): Complete Round 1 results of a randomized trial comparing HPV-based primary screening to liquid-based cytology for cervical cancer. Cancer Ther Prev. 2017;140(2):440-8.
7. Ogilvie GS, van Niekerk D, Krajden M, et al. Effect of screening with primary cervical HPV testing vs cytology testing on high-grade cervical intraepithelial neoplasia at 48 months (The HPV FOCAL randomized clinical trial). JAMA. 2018;320(1):43-52.
8. Statistic Canada. Data quality, concepts and methodology: Definitions. 2012. Available at: https://www150.statcan.gc.ca/n1/pub/84f0210x/2008000/technote-notetech1-eng.htm. Accessed 13 September 2019.
9. McCredie MR, Sharples KJ, Paul C, et al. Natural history of cervical neoplasia and risk of invasive cancer in women with cervical intraepithelial neoplasia 3: a retrospective cohort study. Lancet Oncol. 2008;9(5):425-34.
10. Shumanty R. Report on the Demographic Situation in Canada. Mortality: Overview, 2014 to 2016. Statistics Canada; 2018. Available at: https://www150.statcan.gc.ca/n1/pub/91-209-x/2018001/article/54957-eng.htm. Accessed 13 September 2019.
11. Brodersen J, Schwartz LM, Woloshin SJA. Overdiagnosis: how cancer screening can turn indolent pathology into illness. APMIS. 2014;122(8):683-9.
12. Hamashima C, Hearasawa T, Katayama T, et al. 92 Systematic review of overdiagnosis in cervical cancer screening: how should we define overdiagnosis in cervical cancer screening? BMJ. 2018;23(Suppl 2).
13. van Luijt PA, Rozemeijer K, Naber SK, et al. The role of pre-invasive disease in overdiagnosis: a microsimulation study comparing mass screening for breast cancer and cervical cancer. J Med Screen. 2016;23(4):210-6.
14. Malila N, Leinonen M, Kotaniemi-Talonen L, et al. The HPV test has similar sensitivity but more overdiagnosis than the Pap test - a randomised health services study on cervical cancer screening in Finland. Int J Cancer. 2013;132(9):2141-7.
15. Brodersen J, Siersma V, Thorsen H. Consequences of screening in cervical cancer: development and dimensionality of a questionnaire. BMC Psychol. 2018;6(1):39.
16. The Screening Performance Indicators Working Group, Cervical Cancer Prevention and Control Network. Peformance monitoring for cervical cancer screening programs in Canada. Ottawa, Canada: Public Health Agency of Canada; 2009. Available at: https://www.canada.ca/en/public-health/services/chronic-diseases/cancer/performance-monitoring-cervical-cancer-screening-programs-canada.html. Accessed 13 September 2019.
17. Decker KM, McLachlin CM, Lotocki R on behalf of the Pan-Canadian Cervical Cancer Screening Network Monitoring Program Performance Working Group. Performance measures related to colposcopy for Canadian cervical cancer screening programs: identifying areas for improvement. J Obstet Gynaecol Can. 2015;37(3):245-51.
18. Dulay AT. Cervical insufficiency. 2019. Avaialble at: https://www.merckmanuals.com/en-ca/professional/gynecology-and-obstetrics/abnormalities-of-pregnancy/cervical-insufficiency. Access 13 September 2019.
19. Stedman's Medical Dictionary, 24th edition. Baltimore, Maryland; 1982.
20. Kyrgiou M, Athanasiou A, Kalliala IEJ, et al. Obstetric outcomes after conservative treatment for cervical intraepithelial lesions and early invasive disease. Cochrane Database Syst Rev. 2017;(11):CD012847
21. Nynas J, Narang P, Kolikonda MK, et al. Depression and anxiety following early pregnancy loss: Recommendations for primary care providers. Prim Care Companion CNS Disord. 2015;17(1).
22. Public Health Agency of Canada. Perinatal health indicators for Canada 2017. A report from the Canadian Perinatal Surveillance System. 2017. Available at: http://publications.gc.ca/collections/collection_2018/aspc-phac/HP7-1-2017-eng.pdf. Accesed 13 September 2019.
23. March of Dimes. Low birthweight. 2018. Available at: https://www.marchofdimes.org/complications/low-birthweight.aspx. Accessed 13 September 2019.
