# Supplementary material for: Screening for the prevention and early detection of cervical cancer: protocol for systematic reviews to inform Canadian recommendations
Source: Syst Rev. 2021 Jan 2;10:2. doi: 10.1186/s13643-020-01538-9 (PMC7777363; doi:10.1186/s13643-020-01538-9)
Supplement: Supplementary file 3 — Additional file 3. Methodological Details. [file 13643_2020_1538_MOESM3_ESM.docx]

**Additional File 3. Methodological Details**

**Eligibility criteria**

For all KQs, we will only include studies from Very High Human Development Index countries.^1^ These studies will be most applicable to the Canadian context in which the guideline will be implemented. To be included, studies must have been published in English or French. With the exception of KQ 2 where we have included some restrictions on study eligibility related to verification bias, we will include all eligible studies regardless of risk of bias or reporting quality.

*Key Question 1: Effectiveness and Comparative Effectiveness*

We will include studies published since 1995 of individuals with a cervix, aged 15 years or older, who have been sexually active (as defined by the study authors) and who are not seeking care due to symptoms or are under suspicion of disease by a clinician. In 1995, the first recommendation about HPV testing in Canada was published.^2^ Since that time, recommendations for the use of HPV testing for triage and for primary screening have evolved substantially.^3^ Although the focus is sexually active individuals, we will not exclude studies that do not confirm this before offering screening. The intervention of interest will include any screening strategy using hrHPV tests and/or cytology with subsequent follow-up of abnormal tests, including: primary screening with cytology (conventional or liquid-based); primary screening with hrHPV testing; cytology screening, which if abnormal may be followed by triage with a hrHPV test; hrHPV screening, which if positive may be followed by triage with cytology or another hrHPV test (e.g., full genotyping); or any other relevant test or combination of tests. We will exclude co-testing as a screening strategy, but use data from relevant studies for the individual strategies where suitable. For effectiveness studies, the comparator of interest will be no routine screening. For comparative effectiveness, we will include the following comparators: any other test or strategy, or the same test or strategy with a different screening interval; universal compared with selective or targeted screening (e.g., by starting age); a different method of sample collection (e.g., self-collections vs. provider collection; home vs. clinic-based collection); a different protocol for evaluation of abnormal screening results (e.g., criteria for immediate colposcopy). We will prioritize randomized controlled trials (RCTs). Only if no data from RCTs is identified (per comparison and per outcome) will we include non-randomized studies with a comparator.

*Key Question 2: Comparative Accuracy*

We will include studies published since 1995 of individuals with a cervix, aged 15 years or older, who have been sexually active (as defined by the study authors) and who have no symptoms of cervical cancer. As per KQ 1 we will not exclude studies if sexual activity has not been specified by the study authors. For the comparative effectiveness of HPV tests, we will include studies where any number of the participants have had a recent abnormal cytology result. For other comparisons we will include studies where up to 25% of the participants have had a recent abnormal result. The accuracy of HPV tests in those with abnormal cytology will be relevant to common triage strategies. Because we are interested in the comparative effectiveness of different screening methods, eligible studies must report on two screening tests (i.e., the index test and a comparator test) in all patients (or randomize to two groups), with each compared to the same reference standard. The index tests of interest will include: primary hrHPV testing with HPV nucleic acid tests alone; or hrHPV testing with HPV nucleic acid tests followed by some form of triage (e.g., cytology or HPV testing with partial genotyping for HPV 16 or 18, sequential partial genotyping for HPV 16 or 18 followed by cytology to further triage those positive for HPV 16 or 18). Comparator tests of interest will include conventional or liquid-based cytology, with or without follow-up by hrHPV testing; or hrHPV testing with HPV nucleic acid tests, followed by a different form of triage than in the index test. Outcomes of interest will include: the number and proportion of individuals positive and negative on each test (i.e., the true positives (TP), false positives (FP), true negatives (TN) and false negatives (FN)), and sensitivity and specificity, with the target conditions being to screen for CIN or ICC. To be included, the reference standard test must have been performed before any management of ICC or pre-cancerous lesions based on the index test result (which would bias the findings). We will include observational studies (i.e., prospective or retrospective cohorts, cross-sectional studies) in which all participants receive both the index and comparator tests followed by verification of the conditions using the reference standard in all patients, or in all screening test-positive patients and a sample (e.g., random 10%) of screening test-negative patients. We will also include RCTs where participants were randomized to different screening tests, and either all or a sample of test-negative patients received the reference standard.

*Key Question 4: Relative Importance of Potential Outcomes from Screening*

We will include studies published since 2000 (based on emergence of studies on relevant health-states utilities)^4^. Studies of patients (i.e., individuals 15 years or older with a cervix, or who have had their cervix removed as part of treatment for cervical cancer) as well of the general public will be considered. The exposures of interest will include: experience with a critical outcome(s) related to screening; exposure to clinical scenarios or information about potential critical outcomes and/or estimates of effect of outcome risks from screening; and probability trade-offs or ratings of different potential critical outcomes (e.g., number of biopsies acceptable to prevent one early diagnosis of ICC), whether in the absence or presence of experience of or exposure to information about the outcomes. The comparison of interest will be a different outcome or group(s) of outcomes (considering critical and important outcomes in this review); a healthy state without the outcome; no comparator (if measuring heath state utilities); and no or another intervention (if applicable for interpreting outcome importance).

*Key Question 5: Effectiveness of Interventions to Increase Screening Rates*

We will include studies published since 2000 of individuals with a cervix who meet criteria for cervical cancer screening, but who have never been screened or who have been under-screened (as defined by the authors of individual studies) as compared to current screening recommendations (e.g., for screening interval). The intervention(s) of interest will include: an invitation to self-sample for hrHPV screening, or any other primary care-based intervention aimed at individuals or care providers with the intent to increase screening rates (e.g., screening reminders, educational interventions, provider recommendations, clinician training in cultural competency, patient-provider communication strategies). We will exclude public health outreach strategies and other community-level interventions that could not be implemented in a primary care setting; however, we will include primary care interventions that are implemented alongside or in support of broader public health initiatives (e.g., reminders and invitations). The comparators of interest are no intervention, or standard practice/usual care (as defined by the study authors, and could include invitations or reminders to screen, or other limited forms of intervention like pamphlets or posters). The outcome of interest will be screening or participation rates. We will take a stepwise approach and include RCTs. We will consider including observational cohort studies only if there are no data available from the trials (for any intervention or comparison).

**Method for Integrating Systematic Reviews**

Integrating Systematic Reviews into New Reviews

One or more systematic reviews may exist that align with one or more key questions (KQs) of the reviews undertaken to inform guidelines of the Canadian Task Force on Preventive Health Care. The Task Force and ERSCs have considered the manner in which new reviews conducted for these guidelines can benefit from efficiencies by incorporating existing systematic reviews, while maintaining methodological rigor in their own systematic review conduct, closely aligning existing reviews within their review scope (i.e., inclusion/exclusion criteria), and maintaining consistency with other Task Force Methods. They have based their approach on work conducted by a methods working group composed of investigators from the Evidence-based Practice Center (EPC) program funded by the U.S. Agency for Healthcare Research and Quality (AHRQ).^5,6^ A summary of the way the ERSCs will operationalize the 12 AHRQ recommendations (Box 1) to meet their needs is outlined below. The integration approach differs from situations when “updating” one or a couple existing systematic reviews (i.e., whereby the Task Force would use high-quality review(s), usually without revisions to the reviews’ scope, search for evidence (apart from updating to present), methodological quality/risk of bias assessments, data extraction, or data analysis), or when using one or more reviews “as is” is deemed suitable (i.e., whereby the Task for would use a suitable review without additional searching or synthesis).

Summary of Task Force Approach

The recommendations developed by AHRQ (Box 1) will serve as an overall framework for ERSC reviews, although in most cases existing systematic reviews will be used to build efficiencies in discrete steps within the review process―mainly search and selection of literature, and data extraction―which will not generally include refinement of the scope or data analysis and interpretation. Moreover, we will not in most circumstances include a systematic review itself as a study design for inclusion (unless the intention is to specifically conduct an overview of reviews). The ability to use any given systematic review will largely depend on how it aligns with the Task Force review’s scope (PICOTS). A further primary consideration will be the comprehensiveness of its search strategy and reporting of literature flow. It is important to note that some Task Force reviews need to be complex with multiple stages (e.g., a review of screening effectiveness for patient-important benefits and harms may require including evidence on indirect evidence of test accuracy and treatment) such that existing systematic reviews may exist for one or more discrete stages but not for others. Some key points on the operationalization, and minor revision, by the ERSCs of these recommendations are provided below.

1. **Choosing systematic reviews**: Following the identification of relevant reviews (a search for systematic reviews may be undertaken for some topics), the evidence for each will be mapped to the PICOTS elements and the quality of the review will be assessed (e.g., using the AMSTAR tool which has been evaluated and found effective to discriminate reviews with high and low quality of methods and reporting).^7^ Some of the Task Force KQs may only have a single existing systematic review for possible incorporation, while others may have more than one; if suitable, a decision between systematic reviews will be based on methodological quality, comprehensiveness and quality of its literature search and reporting (e.g., assessed using PRESS checklist), comprehensiveness of reporting on included studies, and the best fit within the Task Force scope and methods. In some cases two or more reviews may be integrated because, together, they capture the full scope of the Task Force KQ(s). Rationale will be provided for choices made.

**Note:** If no review is deemed a good fit for purpose for integration (i.e., de novo process all together appears to be best option) we will at minimum examine available reviews for their search strategies (to ensure that our search strategies are comprehensive) and review their reference lists for identification of studies.

1. **Searching**: Various strategies will be considered. If one or more reviews are fit for purpose (but do not meet criteria for classification as a systematic review update) and cover a scope that is *very similar or broader* than the Task Force topic, we may update the search(es) if the last search date was prior to 6 months before commencing our review. When there are multiple reviews being considered, updating the literature to present may involve a new comprehensive search strategy to identify studies published after the date of the earliest existing review; this may reduce complexities when trying to implement, document, and remove duplicates from multiple searches. Alternatively, if the scope of the existing review(s) is *narrower* (e.g., missing an element in PICOTS) or the search *deemed sub-optimal in some manner* (e.g., missing key terms, additional database viewed as highly relevant) we may re-run the existing review’s search concurrent with an original (e.g., broader) search and remove the citations previously screened for the other review. If more appropriate, we may update the other review’s search and use a new search for the missing PICO element(s) (e.g., one additional intervention) for a longer time period to meet our timeframe. In cases where we feel screening excluded studies lists is appropriate we will also undertake this. Careful consideration will be used to ensure a comprehensive search is conducted regardless of approach taken; moreover, the ERSC librarians will help determine on a case-by-case basis what approach would be feasible for implementation to ensure aims of building efficiencies are possible.
2. **Screening and selection**: We will assess articles included in all relevant reviews (based on full text if necessary) to determine if they meet our inclusion criteria.
3. **Data extraction and methodological quality assessments**: We will consider incorporating the data on study and participant characteristics rather than extracting these data anew; we may also use the review author’s risk of bias assessments if the tools/methods are consistent with Task Force methods. These steps will create efficiencies but because they are dependent on the quality of the systematic review and extent of reporting, the ERSC staff will verify the data on at least 5 to 10% of studies.^5^
4. **Data analysis**: We will consider using quantitative outcome data from reviews (with verification), but will not typically use meta-analyses or quality (GRADE) assessments of existing reviews.
5. **Reporting**: Transparent reporting of all integration steps used will be included in the evidence review report.

**Box 1. Recommendations developed by AHRQ EPCs*^5,6^** *Strength of evidence refers to AHRQ’s slightly modified approach to the GRADE quality of evidence approach

1. Existing reviews should be confirmed as systematic reviews through the application of a minimum set of eligibility criteria. We propose that the minimum eligibility criteria for systematic reviews include an explicit and adequate search, application of predefined eligibility criteria to select studies, risk of bias assessment for included studies, and synthesis of results.

2. Criteria to assess the relevance, in terms of question elements and currency, and quality of existing systematic reviews under consideration for inclusion in reviews should be predefined.

3. The quality of relevant existing systematic reviews should be assessed in an explicit manner with a minimum set of quality criteria that include search of multiple sources, use of a generally accepted tool for risk of bias assessment, and sufficient information to assess the strength of the body of evidence that includes the major domains of risk of bias, directness, consistency, precision, and reporting bias.

4. The risk of bias assessments from the existing systematic review may be used when the review described an explicit process, including the use of a tool or method that is compatible with the approach of the current review and that assessed the key sources of potential bias.

5. We suggest that risk of bias assessment be repeated in a sample of studies from an existing review under consideration for inclusion in a new review to confirm concordance with current review team approach.

6. We recommend that at a minimum, reviews should narratively describe findings of the prior review(s), including the number and types of studies included, and the overall findings.

7. We recommend that newly identified studies be clearly distinguished from studies in the existing review(s) when presented in the narrative and any tables (eg, separate tables).

8. Summary tables should include sufficient information to support ratings for overall strength of evidence, including ratings for individual strength of evidence domains (study limitations, consistency, precision, directness, reporting bias). The strength of evidence ratings should be based on the underlying primary evidence, not the number or quality of existing systematic reviews.

9. Using strength of evidence domains as a framework (study limitations, consistency, precision, directness, and reporting bias), review authors should consider how new evidence would change estimates of effect or ratings for strength of evidence. A new quantitative synthesis (ie, pooled estimate) is needed if new studies would change conclusions or strength of evidence judgements, or to obtain a more precise or more up-to-date estimate.

10. In cases where the existing systematic review(s) did not complete strength of evidence grading for a comparison and outcome of interest, the strength of evidence should be assessed for the body of evidence, considering primary studies from prior review(s) and any new studies identified.

11. In cases where no new studies are added to the body of evidence, the strength of evidence assessment from the existing systematic review may be used if conducted using an acceptable grading approach consistent with current review context. In these cases, we suggest that the overall strength of evidence assessment be reviewed, considering the strength of evidence domains, to confirm consistency with current review team assessments.

12. In cases where new studies are added to the body of evidence, the strength of evidence may need to be reassessed on the basis of all studies/evidence.

**Risk of bias appraisal**

For KQ 1 we will use the Cochrane Risk of Bias Tool (2011 version) and Newcastle-Ottawa Scale to appraise the risk of bias of randomized and non-randomized trials and observational (cohort or case-control) studies, respectively. If we identify relevant before-after and/or interrupted time series studies we will use the suggested risk of bias criteria for Cochrane Effective Practice and Organisation of Care reviews. We will rely on the risk of bias appraisals previously reported in the 2013 Task Force review^8^ as much as possible. In the 2013 Task Force review, the Cochrane Risk of Bias tool and Newcastle-Ottawa Scale were used and the rating for each risk of bias domain, and overall risk of bias was reported in detail for each study. For these studies, one reviewer will assess risk of bias and compare their assessments, by domain, to those previously reported for agreement. A second reviewer will be called upon for input in the case of inconsistencies. Because justification for the ratings were not reported in the previous review, those of the single reviewer will be added. Although the 2018 USPSTF review also reported on the risk of bias in relevant studies, a different tool was used.

For KQ 2 we will use the Quality Assessment of Diagnostic Accuracy Studies-2 (QUADAS-2) tool to assess the risk of bias. The HTA by CADTH (2019)^9^ and systematic review by Arbyn et al. (2018)^10^ both reported domain-specific assessments of risk of bias per study using the QUADAS-2 tool; however, neither reported justification for their decisions. For studies reported in these reviews, one reviewer will perform the risk of bias appraisals and compare their findings to the previous reports. A second reviewer will be called upon for input in the case of inconsistencies. We will report on the single reviewer’s justifications for the domain-level and overall risk of bias ratings.

For KQ 4, we will use the questions proposed by the GRADE working group for appraising the certainty of evidence about the relative importance of outcomes or values and preferences to assess risk of bias for quantitative study designs. Studies will be judged as low, moderate, serious, or critical risk of bias on each of four domains (selection of participants into the study; completeness of data; measurement instrument; data analysis). If it is deemed necessary to adequately capture potential sources of bias, we may add design-specific items to the tool for different study designs. If we identify qualitative studies, we will assess their risk of bias via the Critical Appraisal Skills Programme Qualitative Checklist.

For KQ 5 we will use the Cochrane Risk of Bias tool (2011 version) to assess the risk of bias of RCTs. The Cochrane systematic review by Everett et al. (2011)^11^ used the 2008 version of the tool to assess risk of bias, so we will not use the reported appraisals for studies retrieved from this review. The systematic review by Arbyn et al. (2018)^10^ used the Cochrane Risk of Bias tool (2011 version) to assess the risk of bias in the included studies and reported the domain-specific risk of bias per study, but without justification. For studies retrieved from this review, one reviewer will assess risk of bias and compare their assessments to those in Arbyn et al.’s review^10^ for agreement. A second reviewer will assist in the case of inconsistencies. We will report the single reviewer’s justifications for the assessments. For studies retrieved from the Cochrane systematic review by Everett et al. (2011)^11^ and for new studies identified via the update searches, two reviewers will independently assess risk of bias and reach consensus. A third reviewer will arbitrate if necessary. In the unlikely event that we include observational studies, we will use the Newcastle-Ottawa Scale or Cochrane Effective Practice and Organisation of Care criteria, depending on study design.

**References**

1. United Nations Development Programme. Table 1: Human Development Index and its components. 2013. Available at: <http://hdr.undp.org/en/content/table-1-human-development-index-and-its-components-1>. Accessed 4 June 2020.
2. Johnson K. Periodic health examination, 1995 update: 1. Screening for human papillomavirus infection in asymptomatic women. Canadian Task Force on the Periodic Health Examination. CMAJ. 1995;152(4):483-93.
3. Saraiya M, Steben M, Watson M, Markowitz L. Evolution of cervical cancer screening and prevention in United States and Canada: implications for public health practitioners and clinicians. Prev Med. 2013;57(5):426-33.
4. Ó Céilleachair A, O'Mahony JF, O'Connor M, et al. Health-related quality of life as measured by the eq-5d in the prevention, screening and management of cervical disease: A systematic review. Qual Life Res. 2017;26(11):2885-97.
5. Robinson KA, Chou R, Berkman ND, et al. Integrating bodies of evidence: existing systematic reviews and primary studies. Methods Guide for Effectiveness and Comparative Effectiveness Reviews [Internet]. Rockville (MD): Agency for Healthcare Research and Quality (US); 2015 Feb.
6. Robinson KA, Chou R, Berkman ND, et al. Twelve recommendations for integrating existing systematic reviews into new reviews: EPC guidance. J Clin Epidemiol. 2016 Feb;70:38-44. PMID: 26261004.
7. Foisy M, Hartling L. Challenges and considerations involved in using AMSTAR in overviews of reviews. 22^nd^ Cochrane Colloquium. Hyderabad (India); 2014 Sept 21-26.
8. Peirson L, Fitzpatrick-Lewis D, Ciliska D, Warren R. Screening for cervical cancer: a systematic review and meta-analysis. Syst Rev 2019;2:35.
9. Chao YS, Clark M, Carson E, et al. HPV testing for primary cervical cancer screening: a health technology assessment. Ottawa: Canadian Agency for Drugs and Technologies in Health (CADTH); 2019.
10. Arbyn M, Smith SB, Temin S, Sultana F, Castle P. Detecting cervical precancer and reaching underscreened women by using hpv testing on self samples: Updated meta-analyses. BMJ. 2018;363:k4823.
11. Everett T, Bryant A, Griffin MF, et al. Interventions targeted at women to encourage the uptake of cervical screening. Cochrane Database Syst Rev. 2011;5:CD002834. PMID:21563135.
