# Supplementary material for: Screening for the prevention and early detection of cervical cancer: protocol for systematic reviews to inform Canadian recommendations
Source: Syst Rev. 2021 Jan 2;10:2. doi: 10.1186/s13643-020-01538-9 (PMC7777363; doi:10.1186/s13643-020-01538-9)
Supplement: Supplementary file 4 — Additional file 4. Synopsis of Reviews Used as Sources of Studies of Study-level Data. [file 13643_2020_1538_MOESM4_ESM.docx]

**Additional File 4. Synopsis of Reviews Used as Sources of Studies of Study-level Data**

| **Review** | **Synopsis** |
| --- | --- |
| **Key Question 1** | |
| CTFPHC 2013^1^ | This review used terms and keywords for cervical cancer, HPV, and cervical cancer screening tests to search Medline, Embase, and Cochrane Central from 1995 to April 2012 for the KQs and from 2005 to February 2011 for the Contextual Questions. No geographic filter was applied to the search, but it was limited to studies in English or French. The searches returned 15,145 potentially relevant citations, of which 531 were scrutinized by full text and 27 (representing 24 unique studies) were ultimately included (3 RCTs and 21 observational studies). The authors also searched the reference lists of the 1996 and 2011 USPSTF evidence reviews, but returned no additional relevant studies. |
| USPSTF 2018^2^ | This review investigated the effectiveness of hrHPV testing, with or without cytology, compared with currently recommended strategies. The review was informed by comprehensive searches of Medline, PubMed, PsycINFO, and the Cochrane Collaboration Registry of Controlled Trials. 8 RCTs, 4 cohort studies, and 1 individual patient meta-analysis of five trials were included. |
| **Key Question 2** | |
| CADTH 2019^3^ | The authors aimed to assess the diagnostic accuracy, clinical utility, safety, cost-effectiveness, patients’ experiences and preferences, ethical issues, and implementation issues of HPV testing as a primary screening tool for cervical cancer screening. The authors searched Medline, Embase, the Cochrane Database of Systematic Reviews, Cochrane Central, the Database of Abstracts of Reviews of Effects, and PubMed for reviews published from 2002 to March 2017, and primary studies published after the last date searched for included reviews. Five systematic reviews were identified. Nine RCTs and 11 non-randomized studies that investigated the diagnostic accuracy of self- of clinician-sampled HPV tests compared with clinician-sampled tests (either cytology or another HPV test) were also included. |
| Arbyn et al. 2018^4^ | In this systematic review, the authors aimed to evaluate the diagnostic accuracy of self-sampled compared with physician sampled high-risk HPV assays for the detection of CIN2+. The authors searched Medline, Embase, and Cochrane Central to April 2018. 56 studies were included. |
| **Key Question 5** | |
| Everett et al. 2011^5^ | In this Cochrane systematic review, the authors investigated interventions targeted at women to encourage cervical cancer screening participation. The authors searched the Cochrane Gynaecological Cancer Group Trials Register, CENTRAL, Medline, Embase, and LILACS for studies published up to March 2009. The review included 37 RCTs of invitational and educational interventions, counselling, risk factor assessment, and procedural interventions. All trials included a non-intervention or usual care comparator. The primary outcome was uptake (participation rate). |
| Arbyn et al. 2018^4^ | In this systematic review, the authors investigated whether offering sampling kits to women who were underscreened generated higher response rates vs. sending invitations or reminders. The authors searched PubMed, Embase, and CENTRAL for studies published from 1990 to April 2018. The review included 25 RCTs reporting on screening participation. |

**References**

1. Canadian Task Force on Preventive Health Care. Recommendations on screening for cervical cancer. CMAJ. 2013;185(1):35-45.
2. Melnikow J, Henderson JT, Burda BU, et al. Screening for cervical cancer with high-risk human papillomavirus testing: A systematic evidence review for the US preventive services task force. Rockville, MD: Agency for Healthcare Research and Quality (US); 2018.
3. Chao Y-S, Clark M, Carson E, et al. HPV testing for primary cervical cancer screening: a health technology assessment. Ottawa: CADTH; 2019. (CADTH Optimal Use Report; vol. 7, no. 1b).
4. Arbyn M, Smith SB, Temin S, Sultana F, Castle P. Detecting cervical precancer and reaching underscreened women by using hpv testing on self samples: Updated meta-analyses. BMJ. 2018;363:k4823.
5. Everett T, Bryant A, Griffin MF, et al. Interventions targeted at women to encourage the uptake of cervical screening. Cochrane Database Syst Rev. 2011;5:CD002834. PMID:21563135.
