# Supplementary material for: Screening for the prevention and early detection of cervical cancer: protocol for systematic reviews to inform Canadian recommendations
Source: Syst Rev. 2021 Jan 2;10:2. doi: 10.1186/s13643-020-01538-9 (PMC7777363; doi:10.1186/s13643-020-01538-9)
Supplement: Supplementary file 5 — Additional file 5. Search Strategies for Key Questions 1, 2 and 4. [file 13643_2020_1538_MOESM5_ESM.docx]

**Additional File 5. Search Strategies for Key Questions 1, 2 and 4**

**Search for Key Question 1 in Ovid Medline**

| **#** | **Searches** | **Results** |
| --- | --- | --- |
| 1 | exp randomized controlled trials as topic/ | 135336 |
| 2 | randomized controlled trial.pt. | 504732 |
| 3 | controlled clinical trial.pt. | 93650 |
| 4 | (random* or sham or placebo*).tw. | 1264465 |
| 5 | placebos/ | 34838 |
| 6 | random allocation/ | 102627 |
| 7 | single blind method/ | 28429 |
| 8 | double blind method/ | 157341 |
| 9 | ((singl* or doubl* or trebl* or tripl*) adj25 (blind* or dumm* or mask*)).tw. | 179079 |
| 10 | (rct or rcts).tw. | 46428 |
| 11 | (control* adj2 (study or studies or trial*)).tw. | 472153 |
| 12 | or/1-11 [RCTs] | 1725275 |
| 13 | Epidemiologic studies/ | 8282 |
| 14 | exp case-control studies/ | 1073731 |
| 15 | exp cohort studies/ | 1984245 |
| 16 | Case-control.tw. | 123612 |
| 17 | (cohort adj (study or studies)).tw. | 200630 |
| 18 | Cohort analy$.tw. | 7866 |
| 19 | ((Follow up or followup) adj (study or studies)).tw. | 49544 |
| 20 | (observational adj (study or studies)).tw. | 104046 |
| 21 | Longitudinal.tw. | 240885 |
| 22 | Retrospective.tw. | 519092 |
| 23 | Cross sectional.tw. | 344814 |
| 24 | Cross-sectional studies/ | 325362 |
| 25 | or/13-24 [NON-RCT STUDIES] | 2968491 |
| 26 | (animals not humans).sh. | 4661220 |
| 27 | 25 not 26 [NON-RCT STUDIES WITH ANIMAL FILTER APPLIED] | 2904871 |
| 28 | Cervix Uteri/ | 27273 |
| 29 | Uterine Cervical Neoplasms/ | 74143 |
| 30 | (cervi* adj2 (cancer$ or dysplasia or neoplasm$ or carcinom$ or tumo?r$ or malignan* or premalignan*)).tw. | 63440 |
| 31 | Cervical Intraepithelial Neoplasia/ | 9845 |
| 32 | Uterine Cervical Dysplasia/ | 3758 |
| 33 | Atypical Squamous Cells of the Cervix/ | 254 |
| 34 | Papillomavirus Infections/ or Papillomaviridae/ | 37455 |
| 35 | or/28-34 [MeSH & KEYWORDS FOR CERVICAL CANCER] | 135143 |
| 36 | Cervix Uteri/cy | 1776 |
| 37 | Human Papillomavirus DNA Tests/ | 503 |
| 38 | Papanicolaou Test/ | 6570 |
| 39 | (hpv adj3 (screen* or test*)).tw. | 6816 |
| 40 | ((Pap or Papanicolaou) adj (smear or test* or screening*)).tw. | 8919 |
| 41 | 36 or 39 or 40 [MeSH & KEYWORDS FOR CERVICAL CANCER SCREENINGS] | 16080 |
| 42 | mass screening/ or screen*.ti. | 222418 |
| 43 | "Cytodiagnosis"/ | 16141 |
| 44 | "Early Detection of Cancer"/ | 24547 |
| 45 | vaginal smears/ | 21943 |
| 46 | (vagina* adj5 (smear* or swab*)).tw. | 5704 |
| 47 | (early adj (detection or diagnosis)).tw. | 135594 |
| 48 | DNA probes/ge | 2774 |
| 49 | Papillomavirus Infections/ge or Papillomaviridae/ge | 11458 |
| 50 | cytology.ti,ab. | 50096 |
| 51 | di.fs. | 2541040 |
| 52 | or/42-51 [MeSH & KEYWORDS FOR GENERALIZED SCREENINGS] | 2802984 |
| 53 | 35 and 52 [CERVICAL CANCER AND GENERALIZED SCREENINGS] | 47621 |
| 54 | 41 or 53 [CERVICAL CANCER SCREENINGS, OR CERVICAL CANCER AND GENERALIZED SCREENINGS] | 53010 |
| 55 | limit 54 to yr="2011 -Current" | 18056 |
| 56 | animals/ not (animals/ and humans/) | 4661220 |
| 57 | 55 not 56 [FINAL CONCEPT, ANIMAL AND DATE LIMITS] | 17821 |
| 58 | limit 57 to (english or french) | 16896 |
| 59 | 12 and 58 [FINAL RESULT AND RCTs] | 1567 |
| 60 | 27 and 58 [FINAL RESULT AND NON-RCTs] | 5669 |
| 61 | 60 not 59 [FINAL RESULT AND NON-RCTs, RCTs SPECIFICALLY FILTERED OUT] | 4972 |
| 62 | 59 or 60 [FINAL RESULT, RCTs/NON-RCTs INCLUDED] | 6539 |

**Search for Key Question 2 in Ovid Medline**

| **#** | **Searches** | **Results** |
| --- | --- | --- |
| 1 | Human Papillomavirus DNA Tests/ | 503 |
| 2 | DNA Probes, HPV/ | 1067 |
| 3 | Papillomavirus Infections/di | 5480 |
| 4 | Papanicolaou Test/ | 6570 |
| 5 | Vaginal Smears/ | 21943 |
| 6 | ((HPV* or hrHPV* or Papillomavirus* or Papilloma Virus*) and (triag* or self-sampl* or self-collect* or self-exam* or home-sampl* or home-collect* or home-based or test* or assay* or genotyping or typing or detect* or amplification)).tw,kf. | 26570 |
| 7 | ((Pap or Papanicolaou) adj1 (smear* or screen* or test*)).tw,kf. | 11092 |
| 8 | "Direct-To-Consumer Screening and Testing"/ | 158 |
| 9 | Early Detection of Cancer/ | 24547 |
| 10 | cytology*.tw,kf. | 54627 |
| 11 | (cytodiagnos* or cyto-diagnos*).tw,kf. | 3590 |
| 12 | or/1-11 [HPV/CANCER TESTING] | 119797 |
| 13 | Cervical Intraepithelial Neoplasia/ | 9845 |
| 14 | Uterine Cervical Dysplasia/ | 3758 |
| 15 | Uterine Cervical Neoplasms/ | 74143 |
| 16 | Atypical Squamous Cells of the Cervix/ | 254 |
| 17 | (cervi* adj3 (precancer* or cancer* or neoplas* or dysplas* or dyskaryos* or tumor* or tumour* or malignanc* or carcinoma* or adenocarcinoma* or lesion* or squamous or small cell or large cell)).tw,kf. | 88970 |
| 18 | (cervi* adj5 (ASCC or ASCUS or ASC-US or ASC-H or AGC or AIS or CIN or CINII* or CIN2* or CINIII* or CIN3 or SIL or HGSIL or HSIL or H-SIL or LGSIL or LSIL or L-SIL or low grade or low-grade or mild or equivocal or borderline)).tw,kf. | 7930 |
| 19 | or/13-18 [9] | 109235 |
| 20 | "Sensitivity and Specificity"/ | 344943 |
| 21 | "Limit of Detection"/ | 28834 |
| 22 | ROC Curve/ | 57024 |
| 23 | exp Diagnostic Errors/ | 115743 |
| 24 | False Negative Reactions/ | 17641 |
| 25 | False Positive Reactions/ | 27587 |
| 26 | "Predictive Value of Tests"/ | 200524 |
| 27 | receiver operating characteristic/ | 57024 |
| 28 | predictive value/ | 200524 |
| 29 | "Diagnostic Uses of Chemicals"/ | 92 |
| 30 | kappa.tw,kf. | 91215 |
| 31 | (Sensitiv* and specific*).tw,kf. | 440073 |
| 32 | (false adj2 (positive* or negative*)).tw,kf. | 76266 |
| 33 | ((positive* or negative*) adj2 (predictive or likelihood)).tw,kf. | 71838 |
| 34 | (predictive valu* or validit*).tw,kf. | 274427 |
| 35 | (receiver adj2 operating).tw,kf. | 70760 |
| 36 | (ROC or AUROC* or SROC or HSROC).tw,kf. | 54001 |
| 37 | ((under or over) adj2 curve*).tw,kf. | 60806 |
| 38 | (detect* adj2 (abilit* or rate*)).tw,kf. | 42970 |
| 39 | ((gold* or reference*) adj2 standard*).tw,kf. | 92141 |
| 40 | ((test or diagnos*) adj2 (perform* or accura* or value* or evaluat*)).tw,kf. | 225810 |
| 41 | or/20-40 [ACCURACY] | 1518194 |
| 42 | 12 and 19 and 41 | 6973 |
| 43 | limit 42 to yr="2017 -Current" | 1022 |
| 44 | limit 43 to (english or french) | 975 |
| 45 | letter/ | 1072848 |
| 46 | editorial/ | 525610 |
| 47 | comment/ | 843291 |
| 48 | case reports/ | 2093592 |
| 49 | review/ | 2638447 |
| 50 | or/45-49 [FLUFF FILTER] | 6194998 |
| 51 | 44 not 50 | 904 |
| 52 | animal/ not human/ | 4661220 |
| 53 | (animal* or bat or bats or bovine* or calves or camel* or canine* or cat or cats or chicken* or chimp* or dog or dogs or equine* or feline* or fowl* or goat* or hamster* or horse* or llama* or mice* or monkey* or mouse* or pig or piglet* or pigs or porcine* or poultry* or primate* or rabbit* or rat or rats or rodent* or sheep* or simian* or swine* or veterinar*).ti,jw. | 2541367 |
| 54 | 52 or 53 [ANIMAL FILTER] | 5169797 |
| 55 | 51 not 54 | 903 |

**Search for Key Question 4 in Ovid Medline**

| **#** | **Searches** | **Results** |
| --- | --- | --- |
| 1 | Cervix Uteri/ | 27273 |
| 2 | Uterine Cervical Neoplasms/ | 74143 |
| 3 | Cervical Intraepithelial Neoplasia/ | 9845 |
| 4 | Uterine Cervical Dysplasia/ | 3758 |
| 5 | Papillomavirus Infections/ or Papillomaviridae/ | 37455 |
| 6 | Atypical Squamous Cells of the Cervix/ | 254 |
| 7 | (cervi* adj2 (cancer* or neoplas* or carcinom* or tumo?r* or malignan* or dysplasia)).tw,kf. | 74224 |
| 8 | (HPV or papillomavir*).tw,kf. | 51871 |
| 9 | CIN.tw,kf. | 10281 |
| 10 | (((Pap or Papanicolaou or cervical) adj (smear or test* or screening*)) and (abnormal or result*)).tw,kf. | 8613 |
| 11 | or/1-10 [MeSH & KEYWORDS FOR CERVICAL CANCER] | 153957 |
| 12 | Choice Behavior/ | 32150 |
| 13 | Decision Making/ | 93923 |
| 14 | Patient Preference/ | 8251 |
| 15 | Decision Support Techniques/ | 20084 |
| 16 | (15D* and (HRQoL or QoL or "quality of life" or health-state or health-status or preference)).tw,kf. | 495 |
| 17 | ((analys#s or valuation? or value? or valuing) adj3 (conjoint or contingent)).tw,kf. | 1671 |
| 18 | (choice? adj2 (behavio?r* or discrete or experiment*)).tw,kf. | 6997 |
| 19 | ((choice? or choos* or consent* or decision*) adj1 informed).tw,kf. | 46583 |
| 20 | (EQ?5D or EQ5D or EuroQoL).tw,kf. | 5872 |
| 21 | (health adj2 util*).tw,kf. | 19249 |
| 22 | (utility adj (value* or score*)).tw,kf. | 2779 |
| 23 | HUI?.tw,kf. | 1918 |
| 24 | (Multi*-attribute or multiattribute or multi*-criteria or multicriteria).tw,kf. | 3733 |
| 25 | (pay adj2 (accept* or willing*)).tw,kf. | 6426 |
| 26 | preference*.tw,kf. | 151141 |
| 27 | prospect theor*.tw,kf. | 326 |
| 28 | (SF-12 or SF-36 or SF-6D or SF12 or SF36 or SF6D).tw,kf. | 26142 |
| 29 | standard gamble*.tw,kf. | 850 |
| 30 | (trade off? or tradeoff?).tw,kf. | 29164 |
| 31 | ((choice? or choos* or chose? or decid* or decis*) adj3 (client* or female* or male* or men or patient* or wom#n*)).tw,kf. | 60001 |
| 32 | best-worst.tw,kf. | 336 |
| 33 | or/12-32 [MeSH & KEYWORDS FOR PATIENT PREFERENCE] | 450680 |
| 34 | Patient Acceptance of Health Care/ | 45907 |
| 35 | Patient Participation/ | 25469 |
| 36 | Attitude to Health/ | 83050 |
| 37 | ((attitude* or accept* or participat*) adj3 (client* or person* or people* or individual* or female* or male* or m#n or patient* or wom#n* or user*)).tw,kf. | 112147 |
| 38 | or/34-37 [MeSH & KEYWORDS FOR ACCEPTABILITY] | 249035 |
| 39 | 11 and 33 [PATIENT PREFERENCE FOR CERVICAL CANCER PATIENTS] | 2250 |
| 40 | 11 and 38 [PATIENT ACCEPTABILITY FOR CERVICAL CANCER PATIENTS] | 4216 |
| 41 | animals/ | 6595369 |
| 42 | humans/ | 18436944 |
| 43 | 41 not (41 and 42) | 4661220 |
| 44 | 39 not 43 | 2237 |
| 45 | 40 not 43 | 4216 |
| 46 | limit 44 to (yr="2000 -Current" and (english or french)) [CERVICAL CANCER PREFERENCE, FILTERS APPLIED] | 1851 |
| 47 | limit 45 to (yr="2000 -Current" and (english or french)) [CERVICAL CANCER ACCEPTABILITY, FILTERS APPLIED] | 3477 |
| 48 | 46 or 47 [PREFERENCE OR ACCEPTABILITY FOR CERVICAL CANCER PATIENTS, FILTERS APPLIED] | 4906 |
