# Supplementary material for: Screening for the prevention and early detection of cervical cancer: protocol for systematic reviews to inform Canadian recommendations
Source: Syst Rev. 2021 Jan 2;10:2. doi: 10.1186/s13643-020-01538-9 (PMC7777363; doi:10.1186/s13643-020-01538-9)
Supplement: Supplementary file 6 — Additional file 6. Data Extraction Items for Each Key Question. [file 13643_2020_1538_MOESM6_ESM.docx]

**Additional File 6. Data Extraction Items for Each Key Question**

We will extract characteristics of each included study, as follows:

- **Study**: authors; year; language; publication type; study design; objectives; funding source(s); sampling technique; sample size calculation; start date; end date (all KQs).
- **Participants**: recruitment method; selection (eligibility) criteria; response rate; representativeness of the sample; number enrolled; number allocated to each group (in trials) (including the number who received the reference standard for KQ2); number screened and/or treated; number analyzed; number lost to follow up; baseline characteristics for the subgroups of interest (age; gender identity; sex; immune function (immunocompromised or not); risk behaviours (e.g., early sexual debut, women who have sex with women, individuals who have multiple sexual partners); screening history (under- or never screened, or regularly screened); race; ethnicity; geographic location (country; rural or urban); immigrant or refugee status; socioeconomic status; pregnancy status; HPV vaccination status) (all KQs). Definition of never- and/or under-screened, as reported by the study authors (KQ5).
- **Interventions/exposures:** details of the screening strategy, including the test(s) used, the type of assay (i.e., generic, partial genotyping, full genotyping) and when available: the screening interval; screening approach (i.e., universal, selective/targeted); method of sample collection (self-collection or physician collection, including the setting of collection [i.e., home or clinic]); definition of a positive test (i.e., treatment threshold); protocol for evaluation of abnormal screening results (i.e., criteria for immediate colposcopy; triage strategy) (all KQs). Critical outcome experienced; definition of critical outcome provided by the study authors; clinical scenario or information about potential critical outcomes and/or estimates of effect on outcome risks from screening provided; trade-off scenario provided or outcome ratings solicited; exposure moderators reported (may include differing descriptions or experience of outcomes in terms of stage, treatments received, severity, time since diagnosis, number of outcomes considered, differing estimates of magnitudes of effect from screening) (KQ4). Details of the intervention, including intervention type; description of the intervention; theoretical basis (if reported) (KQ5).
- **Comparators**: definition of “no screening” or “no routine screening”; details of the alternate screening approach (as per interventions/exposures, above) (KQ1, KQ2). Different critical outcome experienced; definition of the different critical outcome experienced (KQ4). Details of the comparator (including no intervention, as per interventions/exposures, above) (KQ5).
- **Outcomes**: definition reported in the study; method of measurement and ascertainment; timing. See Supplementary File 2 and Tables 1-4 in the main document for outcomes of interest for each KQ.
- **Analysis details**: unit of analysis; statistical methods; available within-study subgroup analyses of interest; adjusted analyses; missing data and how these were handled (e.g., imputation, study author contact).
- **Quantitative findings relevant to outcomes of interest**: number of events (or risk ratios, rate ratios, odds ratios, hazard ratios if the number of events are not reported); measure of variance, if relevant (e.g., 95% confidence interval).
